# Learning about informal fallacies and the detection of fake news: An experimental intervention

**DOI:** 10.1371/journal.pone.0283238

**Published:** 2023-03-29

**Authors:** Timon M. J. Hruschka, Markus Appel

**Affiliations:** Psychology of Communication and New Media, University of Würzburg, Würzburg, Germany; University of Oxford, UNITED KINGDOM

## Abstract

The philosophical concept of informal fallacies–arguments that fail to provide sufficient support for a claim–is introduced and connected to the topic of fake news detection. We assumed that the ability to identify informal fallacies can be trained and that this ability enables individuals to better distinguish between fake news and real news. We tested these assumptions in a two-group between-participants experiment (*N* = 116). The two groups participated in a 30-minute-long text-based learning intervention: either about informal fallacies or about fake news. Learning about informal fallacies enhanced participants’ ability to identify fallacious arguments one week later. Furthermore, the ability to identify fallacious arguments was associated with a better discernment between real news and fake news. Participants in the informal fallacy intervention group and the fake news intervention group performed equally well on the news discernment task. The contribution of (identifying) informal fallacies for research and practice is discussed.

## Introduction

In recent years, the phenomenon of *fake news* has gained an unprecedented level of attention around the globe. The phenomenon itself is not entirely new, supposedly journalistic pieces that lacked substance have been published in previous decades and centuries [1]. Yet, the advent of the internet and social media has fueled discussions on the perils of fake news and the measures to take against it. Indeed, fake news stories range among the most popular news items on social media in terms of user engagement (likes, comments, and shares) [2–4]. Given the substantial consequences of misinformation for individuals and societies, combatting the acceptance and spreading of fake news has become one of the most pressing questions for social scientists [5–7]. Theory and research on reasoning suggest that equipping people with cognitive tools that allow them to process incoming information adequately is a promising strategy to reduce the acceptance and sharing of fake news [8–10]. Our focus here is on argumentative fallacies, more specifically on informal fallacies [11, 12]. Philosophers since Aristotle have claimed that recognizing argumentative fallacies might prevent people from falling for dubious claims [13]. We adopted this philosophical assumption and tested it empirically: we developed a brief intervention to increase people’s ability to detect informal fallacies and we examined the downstream consequences on identifying fake news.

### Informal fallacies

Reasoning about arguments can be fallacious in two ways: formally and informally [11]. Formal reasoning fallacies are arguments that are necessarily false because they do not conform to logical standards of deductive reasoning. One prominent example of formal fallacies is the *fallacy of the undistributed middle* [14]:

(1) All humans are animals
(2) All cats are animals
(C) All humans are cats

The problem with this argument is structural: If we changed the structure of premise (2) into: “All animals are cats”, our conclusion (C) would be correct given the premises. Now the term that appears in both premises (i.e., animals) would appear at least once directly after the quantifier “All” which is necessary for this argument to work.

Although much research has been devoted to the study of deductive reasoning and its formal fallacies [15, 16], erroneous real-world argumentation tends to be dominated by informal fallacies: e.g., in politics [17], advertising [18], or in social media postings [19]. Informal fallacies are not fallacious because of their structure but because of their content [11, 12, 20, 21]. Circular reasoning–a prime example for informal fallacies–, for instance, is not deductively invalid, but the argument “The Yeti exists because the Yeti exists” is certainly a fallacy [11]. Why does this argument fail? The claim that “The Yeti exists” is not supported by sufficient evidence: the putative evidence just repeats the claim. Most researchers would agree on this insufficiency being a unifying feature of informal fallacies: informal fallacies are arguments that fail to provide sufficient support for the claim that one is arguing for [12, 20, 22–24]. Still, informal fallacies do persuade a lot of people and they may shape decision-making more than non-fallacious reasoning [25]: informal fallacies are not only fallacious arguments but also arguments that are likely persuasive [24, 26–32].

There are different frameworks for determining what a fallacy is and what is sufficient support for a specific claim (for a review see [11]); of those different approaches, we chose the argumentation scheme approach (ASA) by Walton [12] because it provides particularly clear guidelines for teaching non-experts about informal fallacies [33]. In the ASA, an exhaustive list of argumentative moves is described and then a set of normative questions is collected which must be answered to determine an argument’s strength [11, 12, 20]. If the critical questions belonging to one argumentative move can be satisfactorily answered, a certain argument is strong.

Let’s look at the following example from a speech by Donald Trump in which he attacked Ford for building a plant in Mexico: “[Ford is] going to build a plant [in Mexico], and illegals are going to drive those cars right over the border. Then, they’ll probably end up stealing the car and that’ll be the end of it” [34]. Using the ASA, we can assess this example as a fallacious use of an argumentative move called the *argument from consequence*. We do not want to have people stealing cars (consequence); hence Ford should not build a plant in Mexico (conclusion). For this argument to be valid, however, one has to answer the question: “What evidence, if any, supported the claim that these consequences will (…) occur if A [building the plant] is brought about?” [12, p. 106]. Since there is no evidence to support the alleged causal link from building a plant to people stealing cars, the argument fails: it is a so-called *slippery slope* argument, an invalid distortion of the argument from consequence (for more examples, see S1 in S1 File).

### The current study

Prior research showed that participants’ ability to correctly identify faulty argument structures like the ones shown above contributed to the comprehension and critical evaluation of argumentative texts [35, 36]. These studies, along with previous successful critical thinking interventions [37–42], point at the feasibility of developing a brief intervention to enhance people’s ability to assess informal fallacies.

We therefore hypothesize that informal fallacy assessment skills will be stronger after a learning intervention on informal fallacies than after a more general learning intervention that focused on fake news and illegitimate persuasion attempts online (fake news intervention as control, Hypothesis 1). In our experiment, the dependent variables were assessed seven to ten days after the intervention, as we wished to rule out that our results were based on short-term activation effects.

Multiple studies have shown that people of all ages have problems in evaluating evidence in discourse or news [43–45]. We suggest that the ability to spot and label informal fallacies remedies this shortcoming to a degree: people with a stronger ability in analyzing informal fallacies should be able to decide if putative evidence for a claim in a piece of news is reliable evidence. Additionally, there is content-analytic evidence that informal fallacies co-occur more often with populist claims than non-populist claims [17]. Thus, we expected that the ability to correctly assess informal fallacies is positively associated with the ability to discern real news from fake news. Connecting these assumptions, we assumed an indirect effect of the intervention on news discernment scores, with the ability to spot and label informal fallacies serving as a mediating variable (Hypothesis 2).

Finally, we expected a main effect of the kind of intervention (informal fallacy intervention versus fake news intervention) and hypothesized that our informal fallacy intervention group will be able to discern better between fake and real news than the fake news group (Hypothesis 3).

## Method

### Participants

The required sample size was computed a priori with G*Power [46]. As effect sizes from a similar study concerning inoculation techniques against misinformation showed minimum effect sizes of *d* = .48 [47], we performed a power analysis for *d* = .48, α = .05, power (1-β) = .80. The output showed that we needed a minimum of 110 participants. To account for potential drop-outs and careless responding we recruited a total of *N* = 131 participants. The participants were randomly assigned to one of the two experimental groups (*n*_if_ *=* 65, *n*_fn_ = 66). After excluding 15 participants to ensure good data quality (for exact exclusion criteria see S2 in S1 File), our final sample amounted to 116 participants (*n*_if_ *=* 58, *n*_fn_ = 58). The participants were German undergraduates participating for 1h of course credit. Among the participants, 89 identified themselves as female (78.1%), 24 as male (21.1%), and 1 as non-binary (0.9%). The average age was 21.43 years (min = 18, max = 28, *SD* = 1.94).

### Materials

**Fig 1:**
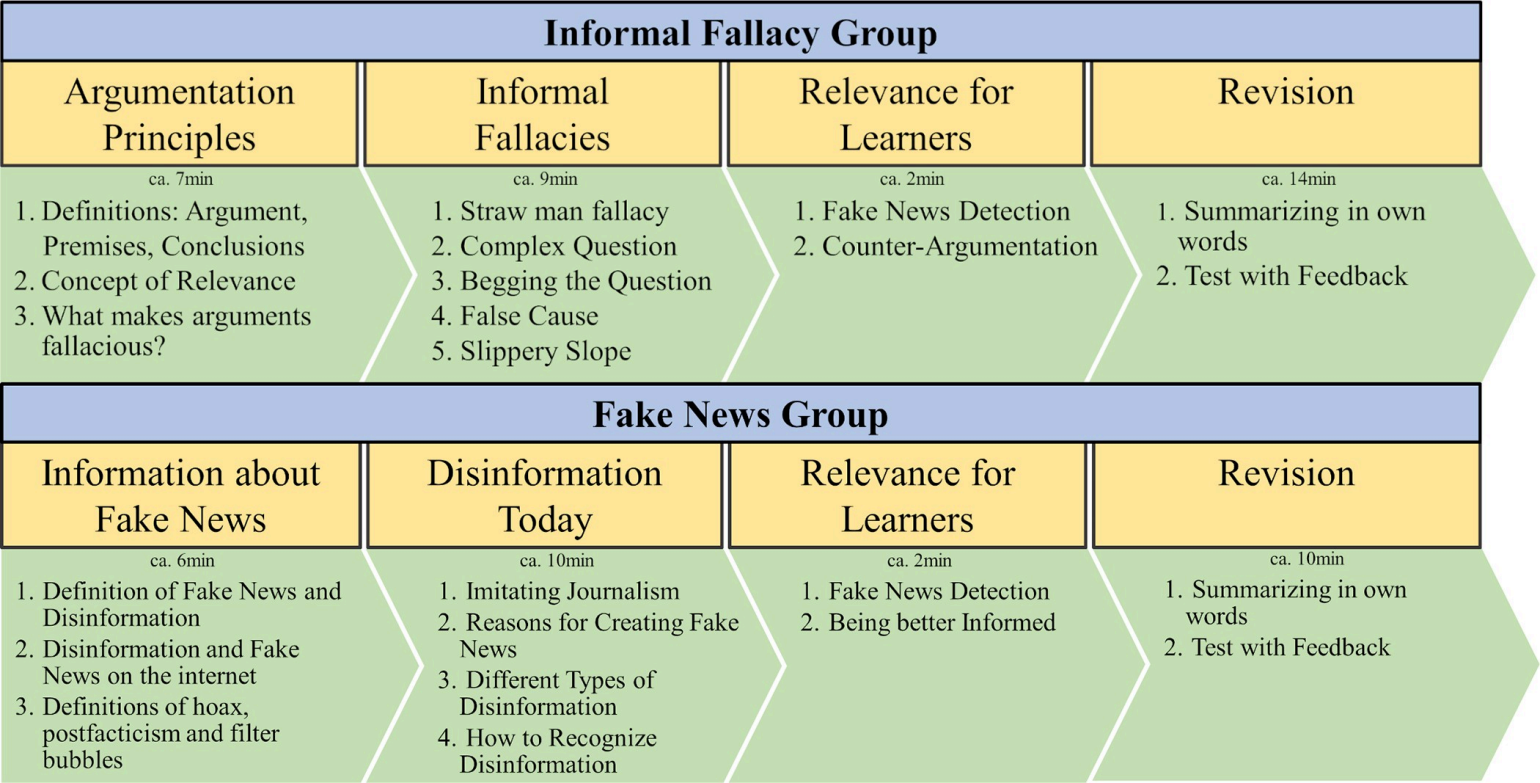
Structure of the two learning interventions. This figure shows the structure of the two learning units including the mean times participants needed to complete a certain part of the unit.

The experimental stimuli consisted of two text-based learning interventions: one on the topic on informal fallacies (informal fallacy group), the other focused on broad information about fake news (fake news group). Both learning interventions were developed by the authors (see Fig 1 and Online Supplements S3 and S4 in S1 File). Each learning session was supervised by the first author to answer potential questions and to ensure diligent work by the participants.

#### Informal fallacy detection intervention

The learning intervention on informal fallacies was based on the ASA by Walton [12] and Tindale [20]. The learning intervention started with eliciting interest in the topic by using an approach developed along the lines of Larson et al. [39]: participants were shown five fallacious arguments asking the participants to tick an argument if they thought it to be fallacious. Only three (of 58) participants correctly assumed all arguments to be fallacious showing most of the participants that there still was much to be learned about.

In the beginning of the following learning intervention, participants were first taught about basic argumentation principles. This includes the concept of *relevance*, that is, a reason must bear some content-related connection to a certain claim in order to support the claim. Afterwards, participants were introduced to five informal fallacies. Each fallacy was introduced by giving an example for the fallacy in question as well as a more detailed analysis of that example. In these detailed analyses participants were shown fallacies with an explanation of why they were fallacious, following approaches in psychological inoculation theory [10, 47–50].

The five fallacies introduced were:

1. *Straw man fallacy*: representing the opponent’s argument in a ‘crooked’ way to make it more easily attackable.
2. *Complex question*: asking a question which, if the opponent would answer it, entails the acceptance of an underlying assertion.
3. *Begging the question*: trying to support a claim by reasons which already assume the claim to be true or repeat the claim in a synonymous way.
4. *False cause*: conclude causality out of mere correlation.
5. *Slippery slope*: designing a causal chain with a negative outcome in which one or more parts of the causal chain do not necessarily follow other parts of the chain.

For every fallacy there were 2–3 questions provided that were meant to help participants to determine if an argument was a fallacy or not [20]. After information about the fallacies was presented, participants read a short paragraph about the importance of knowing about informal fallacies because it could help in identifying fake news. The paragraph did not, however, show the participants how to identify fake news using the concept of informal fallacies. This sets our approach apart from prior inoculation approaches against fake news [47, 48, 51–53]: recent research on psychological inoculation against fake news has focused on showing participants the ways in which *fake news* use misleading strategies. Participants in these studies were exposed to (parts of) fake news. We, however, adopted a broader approach in inoculating participants against *general* reasoning fallacies and tested how that might influence the ability to discern between real and fake news. Participants in the informal fallacy group did not see any fake news during their learning unit.

To deepen the understanding of the fallacies, participants were then asked to write down what a certain fallacy is constituted of in their own words in an open answer field of the survey. Afterwards, participants had to answer nine multiple choice questions (one correct option, four distractors) with immediate feedback telling the participants that their answer was right or how to answer a similar question in the right way if they had answered it incorrectly. Each question dealt with a specific part of the learning intervention. The learning intervention of the informal fallacy group was 3303 words long and yielded a Flesh reading ease of 49.7. Participants needed on average 31.98 (*SD* = 5.97) minutes to complete the informal fallacy group’s learning intervention.

#### Fake news intervention

The fake news learning intervention was structured the same way as the informal fallacy group’s intervention. It started with a portrayal of three fake news asking the participants which of these they believed to be fake. Afterwards, the participants were taught about the phenomenon and the definition of fake news, how to recognize misinformation on the internet, reasons why people create fake news, and about phenomena like filter bubbles and social bots. This was followed by a set of seven characteristics based on which one could determine if a news item was fake [54], resembling the informal fallacy group’s fallacy-identification questions. Afterwards, the participants in the fake news group received nine multiple choice questions, accompanied by similar feedback as the informal fallacy group’s feedback questions. Flesh reading ease for the learning intervention of the fake news group was 40.1 (2776 words). The fake news group needed on average 28.70 *(SD* = 6.23) minutes to work through their learning intervention.

### Measures

#### Informal fallacy task

We used the informal fallacy task (IFT) by Ricco [29] as our main dependent measure. The IFT quantifies the ability to spot and label fallacious arguments correctly. Labeling is in turn linked to the ability to understand the concept of a certain fallacy. The IFT consists of 6 informal fallacies which make up 12 fallacious arguments. We extended the task by adding three non-fallacious arguments that served as filler items.

The ability to spot informal fallacies was measured through a yes or no question asking the participants to indicate if they thought a certain argument to be fallacious. For each correctly spotted fallacy the participants received one point. The ability to label informal fallacies was measured through an open-ended question in which the participants were asked to describe the fallaciousness of the argument or name the exact fallacy. Participants received either 1 point for each fallacy for fully grasping the concept of the fallacy; 0.5 points, if they got one key element right, or 0 points, if their answer indicated that they had not understood the concept of the fallacy in question. The evaluation was performed by the first author using the evaluation criteria by Ricco [29].

Based on the IFT identification and IFT explanation task, we calculated a general IFT score. Before calculating the general IFT score, we excluded three items with negative or near zero factor loadings from the IFT identification task to ensure a better reliability (MacDonald’s ω = .72 before exclusion, ω = .76 after exclusion). The general IFT score was computed by z-standardizing overall IFT identification and IFT explanation scores and averaging the two z-scores for every participant.

#### News discernment task

To measure the discernment accuracy between real and fake news, we presented six real and six fake news items that were developed along the lines of prior work (e.g., [55], for full texts, see Online Supplement S5 and S6 for German texts, S7 and S8 for English translations in S1 File). All news items were presented in random order and consisted of different short news stories portraying a political or scientific topic with their real headlines (e.g., *Did you know*?*—that cancer is an exceptionally rare phenomenon in Israel*? *Why this is so*.). The fake news items were based on actual fake news identified by factchecking websites (*mimikama*.*at* and *correctiv*.*org*) and were on average 125.67 words long (*SD* = 14.22). The real news originated from German quality newspapers (e.g., *Süddeutsche Zeitung*, *Frankfurter Allgemeine Zeitung*), and matched the topic of one of the respective fake news stories (124.17 words on average, *SD* = 19.98). Two news items of each news type (real or fake) were favoring left-wing political narratives, two news items of each type were favoring right-wing political narratives and two news items of each type were politically neutral.

After reading the news, participants were instructed to assess the accuracy of each news item with the help of two questions with options ranging between 1 = *not at all accurate* to 7 = *accurate*, and 1 = *did not happen as described*, 7 = *happened as described*, respectively. To compute the discernment score, we divided the mean of all fake news items through 7 and subtracted the result from the mean of all real news items, also divided through 7. Through this procedure we obtained a value between -1 and 1, where 1 stands for perfect discernment between real and fake news, 0 stands for zero discernment and negative values are assigned to people who believed fake news to be more accurate than real news.

### Procedure

Our study was based on a two-group experimental between-subjects design that took place at two different points in time. At the first point in time, participants worked through the learning interventions. The first session was conducted online, each participant worked on the materials alone. During the first session 2 to 10 participants accessed the materials at the same time and an instructor (the first author of the present manuscript) supervised the learning session to increase compliance and to answer potential questions. The feedback questions were split across the two sessions; in both experimental groups, five of the nine feedback questions were presented in the first session.

Seven to ten days later (mean delay = 7.42 days, *SD* = 0.78 days), the second session took place. The session was not supervised and accessed individually. It started with the remaining four feedback questions referring to the leaning intervention. Then the dependent variable tasks were presented (i.e., the IFT and the news discernment task, S10 in S1 File). Active informed consent was acquired through participation in the online survey. Based on the regulations for conducting psychological research in Germany, no formal IRB approval was required. The studies followed the ethical guidelines of the APA and the German Psychological Society (DGPs).

### Data analysis

We selected a significance level of α = .05 for all statistical analyses and computed two-tailed tests throughout. For effect sizes we computed Cohen’s *d* for *t*-tests and partially standardized effects for our mediation model [56]. As preregistered, outliers (values above or below 1.5 times the interquartile range from the third and first quartile) were identified through boxplot-analyses and were winsorized to one value above or below the last non-outlier [57]. All confidence intervals were based on 10.000 bootstrap-samples. For a more detailed account of our statistical analyses and data quality checks see S11 in S1 File. Zero-order correlations of all measures are presented in Table 1. Data analysis was conducted using *IBM SPSS 27*. Our hypotheses were preregistered using *aspredicted* (small deviations from our preregistration are reported in S12 in S1 File). All data, stimuli, and the preregistration are available at https://osf.io/7ms9e/.

**Table 1:** Zero-order correlations between variables.

	Variable	2	3	4
1	Discernment	.37***	.58***	-.63***
2	IFT general	–	.29**	-.17
3	Real news accuracy		–	.28**
4	Fake news accuracy			–

Pearson correlation. Two-tailed significance tests

*p < .05

**p < .01

***p < .001

## Results

### The informal fallacy learning intervention enhances informal reasoning

A simple regression model including experimental group as predictor and IFT scores as outcome explained 23% of the variance in general IFT scores, *R*^*2*^ = .23, *F*(1, 114) = 33.22, *p* < .001. Participants in the informal fallacy group obtained higher scores in the IFT (M = 0.42, SD = 0.81) than participants in the fake news group (M = -0.41, SD = 0.75, *d* = 1.07). This result was in support of our first hypothesis and indicates that a text-based online learning intervention can foster people’s ability to correctly assess informal fallacies. Cohen’s *d* furthermore indicates a rather strong effect of our learning intervention on the ability to correctly assess informal fallacies.

### The learning intervention’s effects on discernment accuracy are mediated by informal reasoning

In the second hypothesis, we expected that the influence of the intervention on the ability to discern between fake and real news would be mediated by the ability to spot and analyze fallacious informal arguments correctly. To test this hypothesis, we computed a simple mediation model with experimental group as the independent variable (antecedent), the IFT as mediator and the discernment between real and fake news as the criterion (consequent) using *PROCESS 4*.*0 for SPSS* (Model 4; [56]). The hypothesis was then tested by bootstrapping the indirect effect of the antecedent on the consequent–i.e., learning intervention on discernment accuracy–following Hayes [56]. Group membership was dummy-coded (informal fallacy group = 1; fake news group = 0).

**Fig 2:**
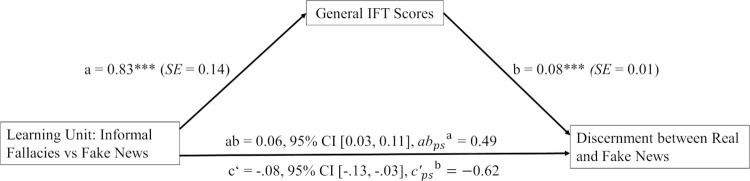
Mediation model with received learning intervention as antecedent, IFT explanation task as mediator and discernment scores as consequent. The graphic shows the mediation model concerning H1 (a), H2 (ab) and H3 (ab+c’). Intervention was dummy-coded with 1 = receiving the informal fallacy intervention and 0 = receiving the fake news intervention. Coefficients presented are unstandardized. For direct and indirect effect no NHST was conducted but instead Bootstrap-CIs are reported. ***p < .001. ^1^ *ab*_*ps*_ = partially standardized effect size for the indirect effect. ^2^ *c’*_ps‘_ = partially standardized effect size for the direct effect.

The indirect effect through informal reasoning was significantly positive, indicating that the learning intervention on informal fallacies enhanced the ability to discern between real and fake news through the ability to correctly assess informal fallacies, estimate = 0.06 (*SE* = 0.02), 95%CI [0.03, 0.11]. The partially standardized effect size was 0.49 (*SE* = 0.13), 95%CI [0.26, 0.77], indicating that if one completed the learning intervention on informal fallacies, one would–on average–be 0.49 *SD* better in discerning between real and fake news because of better informal reasoning than one who completed the fake news group’s learning intervention. These results indicated that the null hypothesis could be rejected. A graphical presentation of the mediation model including all hypotheses can be found in Fig 2.

Note that the direct effect–the effect of group condition on discernment after informal reasoning is partialized out–was also significant, estimate = -0.08 (*SE* = 0.03), 95%CI [-0.13, -0.03]. The partially standardized effect size was -0.62. The negative values indicated that the fake news group also enhanced their discernment between real and fake news through a mechanism we did not measure.

### Discernment accuracy: Informal fallacy versus fake news group

Contrary to our third hypothesis, participants in the informal fallacy group did not perform significantly better in discerning between real and fake news than participants in the fake news group. News discernment scores in the informal fallacy group (*M* = 0.16, *SD* = 0.16) and in the fake news group (*M* = 0.18, *SD* = 0.11), did not differ significantly, Welch *t*(101.26) = -0.70, *p* = .489, *d* = 0.13. This finding suggests that learning about informal fallacies is neither better nor worse than traditional teaching approaches to the topic of fake news at enabling students to distinguish between real and fake news.

### Exploratory analysis: Real news rejection and fake news detection

As an exploratory analysis, we investigated if the fake news group would be more skeptic of all sorts of news after hearing about fake news. After learning about fake news, participants in the fake news group might rate all news as less accurate–regardless of veracity [58]. A mixed ANOVA (between subjects factor: experimental treatment; within-subjects factor: news type) revealed a significant main effect of news type, with real news (*M* = 0.65, *SD* = 0.11) being judged as more accurate than fake news (*M* = 0.48, *SD* = 0.12), *F*(1,114) = 188.86, *p* < .001, η^2^ = .62. Moreover, there was a significant main effect of the experimental treatment, participants who were in the informal fallacy group gave higher accuracy judgments (*M* = 0.58, *SD* = 0.13) than participants in the fake news group (*M* = 0.54, *SD* = 0.13), *F*(1,114) = 6.10, *p* = .015, η^2^ = .05. The interaction between group and news veracity was not significant, *F*(1,114) = 0.37, *p* = .546, η^2^ = .003. The two latter results indicated that participants in the fake news group were slightly more skeptic overall than participants in the informal fallacy group.

## Discussion

Connecting philosophical theory and empirical research on informal fallacies to the topic of fake news detection, our aim was to examine whether a brief online intervention could improve participants’ detection of informal fallacies as well as the discernment between real and fake news. A learning intervention on fake news served as our control condition. As expected, the informal fallacy intervention improved the ability to correctly assess informal fallacies. Moreover, the latter ability (measured with the IFT, Ricco [29]) was positively associated with the discernment between fake and real news. As a consequence, detecting informal fallacies served as a mediator as part of an indirect effect of the informal fallacy intervention on news discernment. However, a residual (direct) effect was also significant, indicating that the fake news intervention had benefits on news discernment as well. This explains a non-significant total effect on news discernment, neither group performed significantly better than the other.

Our findings add to previous research in several ways: Most importantly, we introduced the concept of informal fallacy detection to the debate on fake news and provide evidence that the ability to correctly assess informal fallacies can be improved through a brief online intervention. Second, our results show that the ability to identify informal fallacies is positively associated with discerning real news from fake news. Although our informal fallacy intervention did not outperform a fake news intervention regarding news discernment, additional analyses showed that individuals in the informal fallacy intervention group ascribed higher accuracy overall. Thus, learning about informal fallacies might be effective without raising media skepticism overall [59].

Teaching about informal fallacies can be understood as a specific form of psychological inoculation against misinformation so that a person who encounters misinformation is less likely to fall for it [10, 50]. In psychological inoculation theory, there has been a shift from ‘narrow’ to ‘broad’ inoculation approaches: whereas earlier research on inoculation has focused on specific arguments and topics, later inoculation research is concerned with inoculating people against the *techniques* of misinformation like, in this study, informal fallacies [10]. However, most inoculation approaches today are developed in close alignment with examples of the phenomenon: based on actual misinformation, misleading techniques are identified, and interventions against these techniques are developed [47, 48, 51–53].

In this study, we applied a more theory-guided approach: based on philosophical theory on misleading argumentation techniques, we developed an intervention that inoculates people against misleading forms of incorrect *reasoning*–not specific fake news or conspiracy theories. Building on Hutmacher et al. [8], we propose that there are different approaches to inoculation, some more theory-guided (like increasing statistical or argumentative knowledge), and some more phenomenon-based (like exposure to weakened forms of actual fake news), which all can help to inoculate people against misinformation.

One advantage of our approach of applying philosophical concepts to psychological research is the possibility of embedding the informal fallacy approach to misinformation in school and university curricula. Philosophy, and science in general, has dealt with proper argumentation for centuries [36]. Our study shows that this engagement with correct and incorrect forms of argumentation might also have practical relevance beyond the philosophy classroom.

It follows from our results that interventions that teach how to correctly identify informal fallacies could play a role in mitigating the effects of misinformation. Future research could address several questions in this regard: Are there specific informal fallacies that enhance discernment between real and fake news better than other fallacies? Is it enough to learn about the concept of informal fallacies once or is there a cumulative effect, in that, the more fallacies a person learns, the better the person gets at discerning between real and fake news? Could education on *formal* fallacies also enhance the ability to discern between real and fake news? Does the ability to assess informal fallacies also produce positive outcomes on a societal level, in that, sharing of fake news gets reduced?

As a major limitation, our study did not include an inactive control group. Our control group was active in that participants learned about fake news which was in all likelihood beneficial to news discernment by means other than detecting informal fallacies. To reveal effect sizes of both learning interventions compared to people who did not receive an intervention, research with less active control groups is required. Moreover, our downstream variable was news discernment exclusively. We assume that responses to other post-truth phenomena could profit from detecting informal fallacies as well. For example, the identification of informal fallacies could be applied to detect problems with a net of assertions constituting a conspiracy theory, to unmask bullshitting [60], or to spot shock and chaos disinformation [61].

## Conclusion

The concept of fallacies has guided human thinking on deficient argumentation from early on. In recent studies, the concept of informal fallacies has been applied to the context of news [62, 63]. As informal fallacies seem to be present in many fake news [62, 63], teaching how to recognize and dismantle informal fallacies might be a promising approach to reduce the acceptance and sharing of fake news. This approach has two advantages: First, informal fallacies are domain-independent, meaning that one does not need to be an expert in a particular field to recognize an informal fallacy. A person that might not be an expert on, for example, climate change might still recognize that a certain fake news article is trying to convince them by using complex questions or straw man fallacies, therefore being able to identify the article as not trustworthy. Second, an intervention on informal fallacies could inoculate people against fake news without increasing general media skepticism. A problem of interventions focused more on fake news might be a resulting belief that all news are fake–even real ones [58].

Future research might investigate which other kinds of interventions based on the concept of informal fallacies can reduce the acceptance and sharing of fake news. In our experiment, we employed a text-based online intervention. Interactive classroom interventions in which students discuss real-world examples of informal fallacies could further deepen the understanding of the concept. In addition, very brief online interventions such as social media ads could increase the number of people taught about informal fallacies–reducing the influence of fake news on a larger scale [64]. Future research in this regard might also employ different outcome variables like digital behavior traces on social media. Using social media interventions and tracking the following behavior could reveal more in-depth behavioral patterns like information seeking and sharing behavior [65].

This study shows that a brief online intervention can improve informal reasoning skills. Our research further indicates that increasing citizens’ ability to identify informal fallacies could enable them to better discern fake from real news. We encourage educators to add teaching about informal fallacies to established approaches on teaching about fake news and online misinformation.

## Supporting information

S1 File (DOCX)
