# Supplementary material for: Learning about informal fallacies and the detection of fake news: An experimental intervention
Source: PLoS One. 2023 Mar 29;18(3):e0283238. doi: 10.1371/journal.pone.0283238 (PMC10057814; doi:10.1371/journal.pone.0283238)
Supplement: S1 File — (DOCX) [file pone.0283238.s001.docx]

# **Online Supplement for “Learning about Informal Fallacies and the Detection of Fake News”**

S1 Examples for Informal Fallacies introduced in the Learning Intervention page 2

S2 Participants and Exact Exclusion Criteria page 3

S3 Text of the Informal Fallacy Learning Intervention (German) page 4

S4 Text of the Fake News Learning Intervention (German) page 17

S5 News Discernment Task: Fake News Texts (German) page 29

S6 News Discernment Task: Real News Texts (German) page 33

S7 News Discernment Task: Fake News Texts (English translation) page 36

S8 News Discernment Task: Real News Texts (English translation) page 39

S9 Results for IFT Identification and IFT Explanation Tasks page 42

S10 Detailed Account of Procedure page 44

S11 Additional Information on Data Analyses and Data Quality Checks page 46

S12 Deviations from Preregistration page 47

S13 Exploratory Analysis page 49

Supplemental References page 50

**S1 Examples for Informal Fallacies introduced in the Learning Intervention**

1. *Straw man fallacy*: “Politician A: ‘Why is the government once again moving crime to the top of its agenda when far more pressing issues like childhood poverty (…) continue to be underaddressed?’

Politician B: ‘I am surprised that my honorable friend thinks crime is so unimportant. With the alarming increase in gang violence in our cities we face a breakdown of law and order on a major scale.’” (Tindale, 2007, p. 20)

Here, Politician B does not represent A’s argument in a right way. Politician A never said that crime is an unimportant issue. They just said that there were more pressing issues.

1. *Complex question*: Example: “When are you finally realizing that science is not a way to find the truth?” This example assumes that science is not a way to truth – which is something that should be ascertained first.
2. *Begging the question*: Example: “God is the only perfect being and perfection includes all the virtues. So, we know that God is benevolent” (Tindale, 2007, p. 75). The premise that God is the only perfect being already assumes the claim that God is benevolent, to be true.
3. *False cause: Example*: “COVID-19 is most prevalent in areas with 5G towers. Therefore, 5G causes COVID-19.” Causality does not follow from correlation.
4. *Slippery slope*: An example of a Slippery Slope argument is given in the Introduction section of the main manuscript.

**S2 Participants and Exclusion Criteria**

Of the 131 participants, seven were excluded because they answered some or all parts of the questionnaire much faster than the other participants (Leiner, 2019). If we detected a gap in the distribution of answering speed, we excluded the participants whose duration was below the gap. Two participants were furthermore excluded because they showed multiple strong outliers (more than three times the interquartile range between first and third quartile) in their time needed to answer single questions indicating that they were interrupted multiple times. We did not exclude participants who just stopped the questionnaire once. One participant was excluded due to their self-reported misunderstanding of the tasks they had to perform. One was excluded because of getting help by external resources. Four participants were excluded because they did not seem to answer the questions measuring the dependent variables properly (three had an even-odd consistency in all DVs of less than -.30 after applying the Spearman-Brown-Prophecy formula, one just ticked the middle of the scale in 10 of 12 possibilities). In using answering speed, outliers in answering times and even-odd consistency to determine meaningless answers in online questionnaires, we follow several pieces of advice to identify careless responses in online questionnaires which in turn can have an impact on the study’s results (Leiner 2019; Maniaci and Rogge 2014; Meade and Craig 2012; Patton et al. 2019).

**S3 Text of the Informal Fallacy Learning Intervention**

***1. Aufgabe***

Betrachten Sie folgende Argumentationen. Überlegen Sie, welche dieser Argumentationen Ihnen fehlerhaft und welche Ihnen schlüssig vorkommen. Sie können dabei völlig intuitiv vorgehen. Wenn Ihnen eine Argumentation fehlerhaft vorkommt, markieren Sie diese bitte mit einem ‚F‘ am rechten Rand dieser Seite. Achten Sie auf die Argumente selbst und nicht darauf, ob Sie einzelnen Inhalten aufgrund ihrer eigenen Einstellungen zustimmen oder nicht.

1. In Kanada wurde 2003 die gleichgeschlechtliche Ehe durch ein Gericht zugelassen. Dieses Gerichtsurteil wird unseren Blick auf das Konzept der Ehe für immer verändern. Wenn man mit gleichgeschlechtlicher Ehe anfängt, wird irgendwann auch Polygamie zugelassen werden.
2. Ein Flugzeug, das schwerer ist als Luft, könnte niemals fliegen, denn um abzuheben und über mehrere Meilen fliegend zu können, müsste die Maschine leichter sein als die Umgebung.
3. Ich bin beunruhigt über den Grad an Desinformation, den Fox News täglich unter seinen Zuschauern verbreitet. Als Amerikas beliebtester Kabel-Nachrichtensender trägt er sicherlich einen Teil der Schuld dafür, dass mehr als ein Drittel der Amerikaner immer noch glauben, Saddam Hussein sei für die Anschläge vom 11. September verantwortlich.
4. Mit der Stammzellenforschung stehen wir an der Schwelle zu einer Zukunft, die unermessliche Vorteile in Form von verbesserter Gesundheit und der Beseitigung von Krankheiten verspricht. Doch Kritiker sagen uns, dass wir diesen Weg nicht beschreiten dürfen, weil wir nicht wissen, welche Risiken darin liegen könnten. Was ist das für eine Haltung gegenüber dem wissenschaftlichen Fortschritt? Stellen Sie sich vor, die frühen Weltraumforscher hätten auf all die Pessimisten gehört, die sich über mögliche Risiken Sorgen gemacht hatten. Wir hätten die gewaltige Menge an Wissen, die sich daraus ergeben hat, nicht erhalten.
5. Herr Premierminister, die Zinssätze für Kredite haben sich im vergangenen Jahr fast verdoppelt. Warum unternimmt Ihre Regierung nichts, um Hausbesitzern mit Krediten zu helfen?

Wie viele der auf der ersten Seite dargestellten Argumentationen haben Sie als fehlerhaft markiert? Eine, mehrere, oder alle?

Sie liegen richtig, wenn Sie alle als fehlerhaft markiert haben!

Die folgenden Absätze sollen einer kurzen Erläuterung dienen, warum diese Argumente fehlerhaft sind.

1. Das erste Argument stellt eine Beziehung zwischen gleichgeschlechtlicher Ehe und Polygamie her. Es wird behauptet, Polygamie sei ähnlich zu gleichgeschlechtlicher Ehe und wenn wir gleichgeschlechtliche Ehe zulassen, wird aufgrund dieser Ähnlichkeit (die nicht weiter ausgeführt wird) auch Polygamie zugelassen werden. Dabei wird in diesem Argument allerdings kein Beleg für diese Analogie gegeben und ferner der Umstand übersehen, dass bei der gleichgeschlechtlichen Ehe einer Gruppe erlaubt wird etwas zu tun, was anderen Menschen bereits erlaubt ist. Dies ist bei Polygamie nicht der Fall, sie würde neu eingeführt werden.
2. Das zweite Argument ist ein klassischer Fall von begging the question (was genau das ist, werden wir später noch ergründen). Das Argument versucht zu belegen, dass Dinge, die schwerer sind als Luft, nicht fliegen können, indem es davon ausgeht, dass Dinge, die fliegen können, leichter als ihre Umgebung (und damit als Luft) sind. Das Argument setzt das, was es zu beweisen versucht, bereits voraus.
3. Dieses Beispiel mag auf den ersten Blick recht plausibel klingen, aber es enthält einen Argumentationsfehler namens False Cause, den sie später noch kennenlernen werden. Egal wie viel Schuld man Fox News an der Verbreitung von Desinformation in die Schuhe schieben möchte, in diesem Fall ist das Argument nicht gerechtfertigt. Hier wird ein Zusammenhang zwischen Fox News als größtem Nachrichtensender und dem Glauben von 1/3 der Amerikaner hergestellt, Saddam Hussein sei für die Anschläge von 9/11 verantwortlich. Es gibt allerdings keine Belege für diesen Zusammenhang. Fox News könnte der größte Nachrichtensender sein und trotzdem könnte keine einzige Person, die an Saddam Hussein als großem Verschwörer glaubt, jemals in ihrem Leben Fox News konsumiert haben. Das ist zwar unwahrscheinlich, aber möglich. Da Alternativerklärungen, wie z.B. Desinformation über Social Media (und nicht über Fox News), nicht ausgeschlossen werden können, ist dieses Argument fehlerhaft.
4. Dies ist ein klassischer Fall der Straw man fallacy, die Sie auch später kennenlernen werden. Diejenigen Personen, die heute kritisch gegenüber Stammzellenforschung eingestellt sind, werden gleichgesetzt mit denjenigen Personen, die früher kritisch gegenüber der Weltraumfahrt waren. Damit wird die Position derjenigen, die Stammzellenforschung nicht positiv sehen, nicht akkurat abgebildet, sondern mit einer anderen, leichter anzugreifenden Position gleichgesetzt. Das geschieht ohne Grund für eine solche Gleichsetzung, denn die Argumente von KritikerInnen der Stammzellenforschung und derjenigen der Raumfahrt dürften recht verschieden sein.
5. Zugegeben, dieser letzte Fall ist wirklich ein Grenzgang. Eine Frage ist, wie wir später sehen werden, kein richtiges Argument. Trotzdem werden Fragen, wie auch hier, benutzt, um Menschen von etwas zu überzeugen. Der Trick bei dieser Frage ist, dass etwas vorausgesetzt wird, dem der/die Antwortende vielleicht gar nicht zustimmen würde. Wenn der Premierminister in vorliegendem Fall die Frage beantworten möchte, dann muss er zustimmen, dass seine Regierung nichts für die Hausbesitzer unternommen hat. Das stimmt aber womöglich gar nicht.

***2. Aufgabe: Lerneinheit***

Lesen Sie sich den Text folgender Lerneinheit gründlich und konzentriert durch. Achten Sie auf weitere Anweisungen.

Womöglich haben Sie sich gerade eben gefragt: „Was soll hier fehlerhaft sein?“ Vielleicht sind Ihnen keine wirklichen Fehler begegnet, nur mehr oder weniger überzeugende Argumentationen. Der Begriff der ‚Fehlerhaftigkeit‘ hat Ihnen unter Umständen Schwierigkeiten bereitet.

Sie könnten aber auch bei einigen Argumentationen ein Gefühl gehabt haben, dass hier ‚etwas faul‘ war. Den genauen Fehler in der Argumentation konnten Sie möglicherweise dennoch nicht finden. Oder Sie hatten kein Problem bei der zuvor gestellten Aufgabe, haben alle Fehler richtig identifizieren können und kennen sich in der Verwendung von Argumentationen bereits aus.

Egal was Sie beim Bearbeiten der ersten Aufgabe erlebt haben, die folgende Lerneinheit wird Ihnen dabei helfen, Ihr Wissen um Fehler in Argumentationen zu stärken, zu erweitern und die gelernten Inhalte auf reale Zusammenhänge anzuwenden. Wissenschaftler sind sich einig, dass das menschliche Schlussfolgern ein Werkzeug ist, mit dem Menschen ihre eigenen Überzeugungen, aber auch die Überzeugungsversuche anderer hinterfragen können. Schlüsse zu ziehen und Argumentationen zu bewerten, kann uns so dabei helfen, eine gesicherte Sicht auf die Welt aufzubauen.

Wie die meisten menschlichen Fähigkeiten ist auch das Argumentieren lern- und trainierbar. Da wir es im täglichen Leben oft mehr oder weniger bewusst verwenden, sind die meisten von uns bereits intuitiv gut im Argumentieren. Ohne die aktive Auseinandersetzung mit einem Thema bleibt die Intuition jedoch fehleranfällig.

Die folgende Lerneinheit wird Ihnen zunächst allgemeines Wissen über Argumentationen vermitteln und danach auf einige der wichtigsten Fehler eingehen, die in Argumentationen auftreten können.

**Argumentation allgemein.**

Mit Argumentationen beschäftigt sich u.a. das sogenannte ‚kritische Denken‘. Das Ziel des kritischen Denkens ist es, zwischen guten und schlechten Argumenten unterscheiden zu lernen. Dazu schauen wir uns zunächst folgenden Dialog an:

Anna: „Bitte mach die Tür zu, es zieht. Und wenn es zieht, dann werden wir alle furchtbar krank. Das möchtest du doch auch nicht!“

Tom: „Wenn man ein Immunsystem hat, dann wird man von dem kleinen Zug nicht krank. Daher sehe ich keinen Grund, die Tür zu schließen!“

Anna: „Das ist falsch. Auch mit einem funktionierenden Immunsystem kann man leicht krank werden, wenn es zugig ist.“

Um Argumente analysieren zu können, muss man sie zunächst identifizieren. Der Satz: „Tom, mach bitte die Tür zu!“, ist beispielsweise kein Argument und damit nicht in unserem Sinne analysierbar.

Die Aussage: „Und wenn es zieht, dann werden wir alle krank. Das möchtest du doch auch nicht“, lässt sich jedoch analysieren. Dazu führen wir drei Begriffe ein (Es werden nicht viel mehr Fachbegriffe in dieser Lerneinheit vorkommen, versprochen. Die drei folgenden Begriffe zu kennen, ist allerdings sehr wichtig für die Analyse und Evaluierung von Argumentationen.):

1. Aussagen: Aussagen sind Sätze, die wahr oder falsch sein können. Dabei sind Aussagen nicht unbedingt gleichbedeutend mit Sätzen. Eine Aussage ist eine Art Abstraktion aus einem Satz, etwas, das wir uns mental vorstellen, wenn wir den Satz lesen.
2. Konklusionen: Konklusionen sind Aussagen (s.o.), die eine Behauptung eine/r Teilnehmer/in in einem Dialog gegen Zweifel anderer Teilnehmender verteidigt. Hier wäre es „Und wenn es zieht, dann werden wir alle krank.“
3. Prämissen: Prämissen sind Aussagen (s.o.), die Gründe geben, warum man eine Konklusion annehmen sollte. Hier wäre es z.B. Annas Aussage: „Auch mit einem funktionierenden Immunsystem kann man leicht krank werden, wenn es zugig ist.“. Diese Aussage (sie kann wahr oder falsch sein), unterstützt Annas Konklusion, „wenn es zieht, dann werden wir alle krank.“

Bei Argumentationen ist es sehr wichtig, darauf zu achten, dass nur Aussagen Teil von Argumentationen sein können. Fragen können beispielsweise Teil eines Dialogs sein, nicht aber Teil einer Argumentation. Wenn man eine Aussage trifft, legt man sich darauf fest, dass diese stimmt, man übernimmt Verantwortung. Bei einer Frage ist das nicht der Fall. Sie versucht, das Gegenüber dazu zu bringen, etwas zu präzisieren oder greift eine der Prämissen an, indem sie Zweifel säht. Fragen selbst sind normalerweise keine Aussagen.

**Argumentationsfehler.**

Zunächst soll geklärt werden, was Argumentationsfehler sind und wie man ihnen begegnen kann.

Eine bekannte Definition von Argumentationsfehlern wurde im 20. Jahrhundert von C.L. Hamblin aufgestellt:

„Ein fehlerhaftes Argument (…) ist ein Argument, dass gültig zu sein scheint, es aber nicht ist.“

Nun muss man sich aber fragen: „Wann ist ein Argument nicht gültig?“

Man kann auf allgemeiner Ebene sagen, dass ungültige Argumentationen dann auftreten, wenn Argumentationsmuster verwendet werden, ohne die Umstände zu beachten, in denen sie verwendet werden dürfen. Zum Beispiel darf man nur dann analoges Schließen verwenden (z.B. „Gleichgeschlechtliche Ehe ist wie Polygamie, wenn wir gleichgeschlechtliche Ehe legalisieren, legalisieren wir auch Polygamie“), wenn die beiden Sachverhalte, die man für das eigene Argument verwendet, auch wirklich gleich sind.

Der Kontext, in dem ein Argument verwendet wird, ist sehr wichtig. Unser Ziel beim kritischen Denken muss es sein, sensibel für die Kontexte von Argumenten zu werden und erklären zu können, warum ein bestimmtes Argument in einem bestimmten Kontext fehlerhaft ist.

Dazu werden wir im Folgenden fünf der wichtigsten informalen Argumentationsfehler genauer analysieren. Am Ende jeder Analyse stehen 2-3 Fragen anhand derer wir Argumente leichter auf mögliche Fehler überprüfen können.

Um diese Lerneinheit erfolgreich zu bestehen, sollten Sie sich die Namen der Argumentationsfehler einprägen und die Fragen, die mit ihnen verbunden sind, verstanden haben. Sie müssen die Fragen nicht auswendig lernen, es reicht, diese zu verstehen.

Irrelevante Argumente.

Der erste Argumentationsfehler, den wir kennenlernen werden, ist der sogenannte Straw Man. Dieser dient dazu, eine Position der Gegenseite abzuwehren oder sie zu diskreditieren. Dafür wird ein Straw Man gebaut, der diese Position nicht akkurat abbildet, sondern nur eine Karikatur dieser Position ist. Die Karikatur wird gebaut, um den Gegner leichter angreifen zu können. Daher kommt auch der Name des Straw Man, denn es wird eine Strohpuppe erschaffen, um sie leicht anzünden zu können.

Für Sie als Analysierende eines Arguments ist es wichtig zu beachten, dass diese Strohpuppe niemals ganz der eigentlichen Position des Angegriffenen entspricht.

Ein Beispiel kann dies veranschaulichen:

Politiker A: „Warum nennt die Regierung die Kriminalität als oberste Priorität, wenn es aktuell dringendere Themen wie die Kindheitsarmut gibt, um die sich niemand kümmert?

Politiker B: „Ich bin überrascht, dass sich mein werter Freund denkt, dass Kriminalität so unwichtig ist. Mit dem Zuwachs von Bandenkriminalität in den Großstädten könnten wir einen großen Einbruch von Recht und Ordnung erwarten. Politiker A möchte, dass wir in einer Gesellschaft leben, in der Menschen sich nicht mehr in ihren eigenen vier Wänden sicher fühlen können.“

Dieses Beispiel macht den Kern des Straw Man Fehlers deutlich. Politiker B stellt Politiker A’s Aussage anders dar, als sie eigentlich getroffen wurde. Politiker A hat nie gesagt, dass Kriminalität unwichtig ist. Anstatt sich mit dem eigentlichen Argument auseinanderzusetzen, nämlich, dass es dringendere Themen als die Kriminalität gibt, erschafft B einen Straw Man, nämlich dass A angeblich Kriminalität nicht für wichtig hält.

Ein weiteres wichtiges Konzept des kritischen Denkens, dass sich am Beispiel dieses Fehlschlusses zeigen lässt, ist das der Relevanz. B bringt eine irrelevante Aussage ins Spiel. Warum ist sie irrelevant? Weil sie uns keinen Grund gibt, der Aussage von A nun anders gegenüberzustehen. Relevanz heißt, dass eine Prämisse etwas mit dem Kontext, in dem sie getroffen wurde oder mit der Konklusion, die von ihr gestützt wird, zu tun haben muss. In diesem Fall ist die Aussage von B irrelevant für den Kontext, in dem sie getroffen wird. Sie gibt uns keinen Grund, die Aussage von A neu zu bewerten, da sie nichts mit dieser zu tun hat.

Zwei Fragen können uns helfen, Argumentationen nach dem Straw-Man Fehler abzusuchen:

1. Wurde die Position des Gegenübers falsch repräsentiert?
2. Bot diese falsche Repräsentation den Grund für einen Angriff auf das ursprüngliche Argument (oder die Position) des Gegenübers?

**Versteckte Annahmen.**

Dass Fragen keine Aussagen sind und damit auch kein Teil von Argumenten sein können, haben wir oben bereits festgestellt. Trotzdem werden Fragen manchmal zur Stützung einer Konklusion benutzt, was ein Argumentationsfehler ist. Diese sogenannten Complex-Questions arbeiten damit, dass sie implizite Aussagen transportieren. Diesen Aussagen stimmt jede/r, die/der diese Frage versucht zu beantworten, automatisch durch die Beantwortung zu. Ein Beispiel kann dies veranschaulichen:

„Wann wachst du endlich auf und siehst, was um dich herum passiert?“

Diese Frage hat mehrere mitschwingende Aussagen: Erstens beschuldigt sie die befragte Person, blind zu sein für das, was ‚um sie herum passiert‘. Zweitens zwingt die Frage jede/n Antwortende/n dazu, zuzustimmen, dass um ihn/sie herum irgendetwas passiert, obwohl gerade das vielleicht zur Debatte steht.

Complex-Questions treten meist in der Form von „Wann hört XY damit auf, Hunde zu schlagen (Banken auszurauben, Lobbyismus zu betreiben, Morde zu begehen…).

Es gibt zwei einfache Fragen, mit denen man aufdecken kann, ob ein Fall einer Complex-Question vorliegt:

1. Birgt die Frage versteckte Annahmen, die den Antwortenden in eine unakzeptable Situation bringen?
2. Führt das Beantworten der Frage direkt dazu, dass ein unfairer Wechsel der Beweispflicht entsteht? (Im Beispiel hätte eigentlich die/der Fragesteller/in die Pflicht zu beweisen, dass XY Hunde schlägt (Banken ausraubt, Lobbyismus betreibt, Morde begeht…) Nur durch die Fragestellung ist plötzlich die/der Antwortende in der Position beweisen zu müssen, dass er/sie das nicht tut!)

Ein weiterer Trugschluss, der von versteckten Annahmen ausgeht, ist begging the question. Begging the question tritt immer dann auf, wenn die Prämissen, die zur Stützung einer Konklusion herangezogen werden, die Konklusion bereits enthalten. Ein Beispiel dafür haben Sie bereits in der ersten Aufgabe kennengelernt (Argument 2). Ein weiteres Beispiel wäre das folgende Argument:

„Gott ist das einzig perfekte Wesen und Perfektion beinhaltet alle Tugenden. Daher wissen wir, dass Gott gütig ist.“ Hier wird versucht zu beweisen, dass Gott gütig ist. Die Prämisse nimmt bereits die Konklusion an, die aber erst durch die Prämisse bewiesen werden soll. Hier nimmt die Prämisse an, dass Gott alle Tugenden hat und damit per Definition gütig ist. Die Gütigkeit Gottes soll ja aber erst bewiesen werden.

Die folgenden zwei Fragen können uns helfen, aufzudecken, ob ein Argument durch begging the question fehlerhaft ist. Dabei muss nur eine der beiden Fragen zutreffen

1. Hat der/die Argumentierende die Pflicht verletzt, unabhängige Prämissen für seine Konklusion anzugeben, indem die Konklusion nur in einer anderen sprachlichen Form wiedergegeben wird? (z.B. „Bilderbücher sind eine gute Lektüre für Kinder, da für Kinder vor allem Lesestoff mit vielen Bildern gut ist“)
2. Hat der/die Argumentierende die Pflicht verletzt, unabhängige Prämissen für seine Konklusion anzugeben, indem in den Prämissen vorausgesetzt wird, dass die Konklusion stimmt?

**Die Verwechslung von Korrelation und Kausalität.**

Menschen schließen gerne von Korrelation auf Kausalität. D.h. weil Dinge scheinbar zusammenhängen oder gleichzeitig auftreten (=Korrelation), muss das eine das andere herbeigeführt haben (=Kausalität). Diese Art der Argumentation kann richtig sein, aber nur, wenn man bei der Argumentation die folgenden Bedingungen beachtet: 1. Müssen zwei Ereignisse immer zusammenhängen (=Korrelieren) 2. Muss ein Ereignis vor dem anderen eintreten 3. Alternativerklärungen für den Zusammenhang beider Ereignisse müssen ausgeschlossen werden können.

Falls diese Bedingungen nicht gegeben sind und trotzdem von einer Korrelation auf eine Kausalität geschlossen wird, liegt ein Argumentationsfehler vor. Dieser wird häufig als False Cause Fehler bezeichnet. Ein Beispiel kann dies veranschaulichen:

„Die Entscheidung einer großen Zahl von Frauen in den letzten Jahrzehnten, das Haus zu verlassen und stattdessen Karriere zu machen, hat zu einem Rückgang der Geburtenrate geführt, da weniger die Entscheidung treffen, zu Hause zu bleiben und Mütter zu sein.“ Hier liegt eine Korrelation vor, es bleiben tatsächlich immer weniger Frauen dauerhaft zuhause und auch die Geburtenrate sinkt ab. Prinzipiell macht auch die zeitliche Vorgeordnetheit Sinn.

Man könnte sagen, dass einige Frauen aufgrund ihres Jobs keine Kinder bekommen möchten. Das große Problem in dieser Argumentation liegt in der Alternativerklärung: Beide Effekte könnten einfach durch die Fortschritte in Verhütungsmethoden zustande gekommen sein. Jetzt heißt es für Frauen nicht mehr Karriere oder Kind, sondern beides kann besser geplant und in Einklang gebracht werden. Karriere als Kausalgrund für Kinderlosigkeit zu postulieren, ist ein Argumentationsfehler.

Folgende 3 Fragen können uns helfen, False Cause zu erkennen und zu vermeiden:

1. Liegt eine datenbasierte (wirklich gemessene) Korrelation vor?
2. Liegt ein Ereignis wirklich dauerhaft vor einem anderen Ereignis?
3. Können Alternativerklärung für die angenommene Kausalität ausgeschlossen werden?

Das letzte fehlerhafte Argument in dieser Lerneinheit, das wir betrachten wollen, ist das slippery slope Argument. Dieses folgt der einfachen Form: „Wenn X passiert, wird auch Y passieren und Y wollen wir nicht. Daher dürfen wir X nicht passieren lassen.“ Das Argument wird slippery genannt, da nach X auch wirklich Y eintreten muss. Ein Beispiel kann das illustrieren: „Wenn wir den Marihuanakonsum legalisieren, werden irgendwann alle Menschen auch Crystal Meth zu sich nehmen. Daher dürfen wir Marihuana nicht legalisieren“ Damit dieses Argument wirklich gültig ist, muss der Crystal Meth Konsum auch notwendigerweise aus dem Marihuanakonsum folgen. Ansonsten hat die Prämisse (Marihuanakonsum führt zu Crystal Meth Konsum) keinen relevanten Zusammenhang mit der Konklusion (Marihuana darf nicht legalisiert werden). Da dies hier offensichtlicherweise nicht der Fall ist, liegt ein Argumentationsfehler vor.

Folgende drei Fragen können uns helfen, Fehler der Art slippery slope aufzudecken:

1. Sind die postulierten Kausalzusammenhänge plausibel?
2. Ist der Zusammenhang wirklich slippery, oder könnte nach einem der Schritte in der Kausalkette auch einfach Schluss sein?
3. Ist das Ergebnis der Kausalkette wirklich negativ?

**Warum das Ganze?**

Sie haben nun viele Informationen über argumentative Fehlschlüsse erhalten. Doch mögen Sie sich fragen, wozu Sie das ganze lernen sollen.

Wissenschaftliche Forschung konnte zeigen, dass PopulistInnen gerne auf fehlerhafte Argumente zurückgreifen, um Andere von ihren Ansichten zu überzeugen. Es konnte ferner gezeigt werden, dass in durch PopulistInnen imitierten Nachrichten mehr argumentative Fehler vorkommen als in unabhängigen Nachrichten. Das ist z.B. bei Fake-News der Fall, die unabhängige Nachrichten imitieren, dabei aber Lügen verbreiten, um politische oder wirtschaftliche Ziele durchzusetzen.

Natürlich enthalten nicht alle Fake News Argumentationsfehler. Das heißt, nur weil eine Nachricht keine fehlerhafte Argumentation enthält, muss sie noch nicht wahr sein. Eine blanke Lüge wie „Hillary Clinton isst im Keller eines Pizzarestaurants Kinder auf, um ewig jung zu bleiben“ enthält keine Argumentation und damit auch keinen Argumentationsfehler. Trotzdem können wir mithilfe unseres Wissens um Argumentationen vor dem Hintergrund unserer Weltanschauung fragen, ob diese Lüge denn Sinn macht. Wir werden schnell Gegenargumente finden wie: „Hillary Clinton sieht eher alt aus“, & „Aus dem Wissen des Biologieunterrichts, den ich in der Schule hatte, erscheint mir eine Verjüngungskur durch Kinderblut sehr unwahrscheinlich.“ Dadurch stellen wir fest, dass die Beweislast bei dem/der Aufstellenden dieser Lüge liegt. Wenn Diese/r uns jedoch keine Argumente oder Belege zur Stützung dieser These liefert, werden wir sagen müssen: „Eine ‚interessante‘ Vermutung, jedoch erscheint mir das sehr unplausibel, daher kann ich meine Sicht auf die Welt nicht ändern. Es sei denn, es werden neue, plausible Begründungen für diese These dargelegt!“

Das Wissen um Argumentationsfehler ist ein Werkzeugkasten, mit dem wir Nachrichten und Überzeugungsversuche anderer Art evaluieren können. Auf der nächsten Seite finden Sie daher noch einmal eine Übersicht über alle Argumentationsfehler, die Sie in dieser Lerneinheit kennengelernt haben. Um das gelernte auch tiefer zu verstehen lohnt es sich meist, eigene Notizen anzufertigen. Daher bitten wir Sie, zu jedem Argumentationsfehler mindestens einen Satz oder mehrere Stichpunkte aufzuschreiben, die Ihnen dabei helfen, sich besser an den zugrundeliegenden Fehler zu erinnern.

Das können die Fragen sein, die Ihnen helfen sollen, Argumentationen zu überprüfen, das können auch Stichpunkte sein wie:

**S4 Text of the Fake News Learning Intervention**


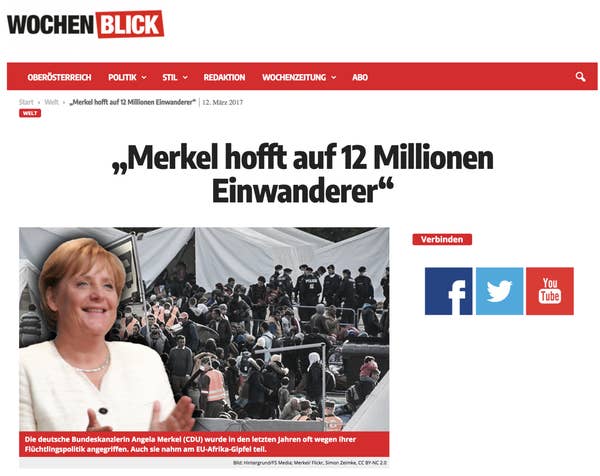
***1. Aufgabe***


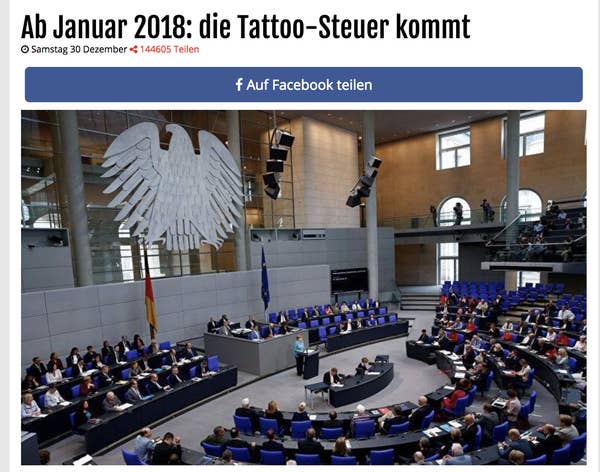

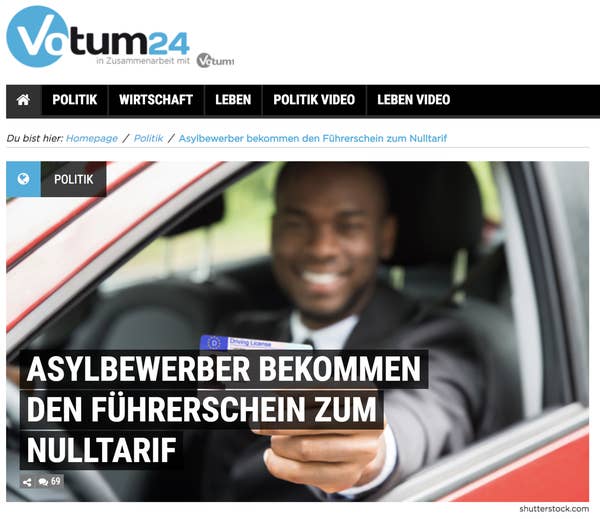
Betrachten Sie die obenstehenden Nachrichten. Überlegen Sie, welche dieser drei Nachrichten ihnen fehlerhaft vorkommt und welche Ihnen ‚real‘. Wenn Ihnen eine Nachricht fehlerhaft vorkommt, streichen Sie diese bitte durch!

Wie viele der auf der ersten Seite dargestellten Nachrichten haben Sie als fehlerhaft markiert? Eine, mehrere, oder alle?

Sie liegen richtig, wenn Sie alle als fehlerhaft markiert haben!

Die folgenden Absätze sollen einer kurzen Erläuterung dienen, warum diese Nachrichten fehlerhaft sind.

„Asylbewerber bekommen den Führerschein zum Nulltarif“

Die Webseite „Votum24.de“ behauptet in diesem Artikel, dass Asylbewerber Führerscheine kostenlos bekämen. Das stimmt nicht.

Autor Dimitris Kazarnovskis schreibt, dass die Zahl der Asylbewerber in Fahrschulen gestiegen ist. Das stimmt. Er schreibt aber auch, dass das Jobcenter die Kosten für Führausbildungen übernehmen würde. Das stimmt nicht.

„Asylbewerber bekommen natürlich keinen Führerschein zum Nulltarif”, sagte Paul Ebsen, Pressesprecher der Bundesagentur für Arbeit in Nürnberg, telefonisch gegenüber BuzzFeed News. Was im Artikel von „Votum24“ nicht erwähnt wird: Es gibt tatsächlich eine Förderung für Führerscheine – aber nur unter bestimmten Bedingungen. Wenn sich ein Arbeitgeber dazu bereiterklärt jemanden zu beschäftigen und für diesen Job unbedingt ein Führerschein benötigt wird, dann kann das Jobcenter die Kosten für den Führerschein übernehmen, erklärte Ebsen gegenüber BuzzFeed News. Diese Förderung sei allerdings nicht auf Asylbewerber beschränkt, sondern gilt für jeden, auch für Deutsche.

„Ab Januar 2018: die Tattoo-Steuer kommt“

Das Onlineportal „24aktuelles.com“ veröffentlichte 2017 den Artikel „Ab Januar 2018: die Tattoo-Steuer kommt“, der insgesamt über 70.000 Interaktionen auf Facebook erreicht hat. Aber der Artikel ist frei erfunden.

Auf dem Portal 24aktuelles.com kann jeder seine eigene Fake News (oder wie die Macher der Webseite es nennen: „Witz“) erstellen — kurzum: Auf 24aktuelles.com finden sich ausschließlich Falschnachrichten.

Die Macher der Seite beschreiben ihr Portal so:

„24aktuelles.com ist eine Internetseite die zur Unterhaltung dient, die falschen Witze werden von unseren Usern verfasst. Alle Witze dieser Seite sind frei erfunden und fiktiv, es ist alles nur Spaß! Keine der Witze sollte ernst genommen werden oder als seriöse Informationsquelle benutzt werden.

„Merkel hofft auf 12 Millionen Einwanderer“

Der Artikel der österreichischen Zeitung „Wochenblick.at“ „Merkel hofft auf 12 Millionen Einwanderer“ erreichte rund 51.000 Facebook-Interaktionen.

Laut des Artikels von „Wochenblick.at“ sind englische Medien auf ein „geheimes Papier“ der Bundesregierung gestoßen, nachdem Angela Merkel hoffe, dass es bis 2060 zwölf Millionen neue Einwanderer in Deutschland gäbe.

Spiegel Online hat ein Debunking zu dieser Falschnachricht geschrieben und hier ausführlich erklärt, was in Wirklichkeit dahinter steckt. Unter anderem schreibt Spiegel Online: „Die Bundesregierung vertuscht keinen Flüchtlingsplan, sondern hat die langfristige Bevölkerungsentwicklung für Deutschland schätzen lassen.”

Außerdem sei das Papier weder geheim, noch hätte es ein „Schweigen der Medien“ über dieses Papier gegeben, so Spiegel Online weiter.

***2. Aufgabe: Lerneinheit***

Lesen Sie sich den Text folgender Lerneinheit gründlich und konzentriert durch. Achten Sie auf weitere Anweisungen.

Womöglich haben Sie sich gerade eben gefragt: „Was soll das? Warum sollten diese Nachrichten fehlerhaft sein?“ Vielleicht ist Ihnen auch gar nicht klar gewesen, woran man diese Fehlerhaftigkeit festmachen soll. Sie könnten auch bei einigen Nachrichten einfach ein Gefühl gehabt haben, dass hier ‚etwas faul‘ war. Den genauen Fehler in der Nachricht konnten Sie möglicherweise dennoch nicht finden. Oder Sie hatten kein Problem bei der zuvor gestellten Aufgabe, haben alle Fehler richtig identifizieren können und kennen sich in der Identifikation von Fake News bereits aus.

Egal was Sie beim Bearbeiten der ersten Aufgabe erlebt haben, die folgende Lerneinheit wird Ihnen dabei helfen, Ihr Wissen um Fake News zu stärken, zu erweitern und die gelernten Inhalte auf reale Zusammenhänge anzuwenden. Doch warum sollten Sie das tun?

Manipulationen durch Social Bots bei Wahlen weltweit, Bildmanipulationen wie das berüchtigte Flüchtlings-Selfie mit der Bundeskanzlerin, das immer wieder in faktisch falschen Kontexten in Sozialen Netzwerken auftauchte und Hetzkampagnen aus der rechtsextremen Szene, die sich gezielter Falschmeldungen über Geflüchtete bedienen: Fake-News – bewusst gestreute Falschmeldungen – stellen eine wachsende Gefahr für die Demokratie dar.

Gerade Jugendliche und junge Erwachsene informieren sich häufig in Sozialen Netzwerken oder Blogs über aktuelle Ereignisse. Dabei laufen sie besonders Gefahr, Falschmeldungen von manipulierenden Nachrichtenseiten aufzusitzen, die dort geteilt werden. Deren Betreiber setzen auf eine perfide Methode: Auf den ersten Blick kaum zu erkennen, machen sie unter dem Deckmantel der seriösen Berichterstattung systematisch Stimmung insbesondere gegen Fremde und Flüchtlinge und locken auf populistische Angebote.

**Das Internet – eine Fundgrube für Hassseiten, Verschwörungstheorien und Falschmeldungen.**

Im Internet verbreiten sich Falschmeldungen (Fake-News oder Hoaxes) als Text-, Bild- oder Videoformat besonders schnell und werden oft unreflektiert geteilt und kommentiert. Dabei wird Fake News als eine gefälschte Nachricht definiert, die nicht mit Satire zu verwechseln ist. Es handelt sich um gezielte Falschmeldung, die auch echte Nachrichtenelemente enthalten kann, auf die sie sich stützt bzw. mit der sie Seriosität vortäuschen möchte. Sie ist in ihrem Stil an echte Nachrichten angelehnt und wird meist über die sozialen Netzwerke verbreitet. In vielen Fake-News wird der Kontext einer Meldung bewusst ausgeblendet oder einzelne Inhalte daraus überspitzt, Fotos und Videos manipuliert oder aus dem eigentlichen Zusammenhang gerissen. Das Ziel solcher Falschmeldungen ist es, mit reißerischen Schlagzeilen und Bildern eine möglichst hohe Anzahl an Klicks zu generieren oder Meinungen in eine gewünschte Richtung zu manipulieren. Solche Fake-News erzielen eine enorme Reichweite, sodass die manipulierten Informationen immer wieder in den Timelines Sozialer Netzwerke auftauchen und ihnen stetig mehr Menschen Glauben schenken. Falschmeldungen werden immer professioneller aufbereitet, und selbst Journalisten fällt eine Unterscheidung manchmal schwer. Anbieter von Falschmeldungen platzieren sie weit vorne in Suchmaschinen, um Werbegelder zu verdienen. Falschmeldungen werden auch als Hoax oder Hoaxmeldung bezeichnet. Laut Wikipeda wird das Wort Hoax von Hocus abgeleitet, welches wiederum eine Verkürzung von Hocus Pocus („Hokuspokus“) ist. Auf der Seite http://hoaxmap.org werden deutschlandweit Gerüchte und Falschmeldungen über Asylsuchende gesammelt und entlarvt. Eine Qualitätskontrolle und die Verifizierung von Informationen wird daher immer schwieriger und die Überprüfung der Inhalte und Quellen unweigerlich wichtiger. Fast bezeichnend für all diese Entwicklungen ist das Wort des Jahres 2016: „postfaktisch“. Das Kunstwort verweist darauf, dass es in politischen und gesellschaftlichen Diskussionen heute zunehmend um Emotionen anstelle von Fakten geht. Die Begriffe „postfaktisch“ und „Fake News“ stehen seit 2017 im Duden. Sie sind somit fester Bestandteil unserer Sprachwelt und leider auch unserer gesellschaftlichen Realität.

**Social Bot.** Der Begriff „Bot“ ist von dem Wort „robot“ abgeleitet. Im technischen Umfeld wird darunter zumeist ein Programm verstanden, welches ohne menschlichen Eingriff Aktionen ausführt. Social Bots sind laut einer Studie der Universität Duisburg-Essen in der Lage, in sozialen Medien Stimmungen zu manipulieren.

**Die Merkmale aktueller Desinformation.**

Will man die Auswirkungen von aktueller Desinformation auf zum Beispiel Wahlen analysieren, sollte man eine sehr genaue Vorstellung davon haben, was man eigentlich untersuchen will. Wie schon am Namen ersichtlich, reden wir von einem Untertypus von Desinformation. Wenn wir im Folgenden dessen Merkmale aufzählen, nehmen wir die komfortable Position eines Beobachters ein, der über genügend Zeit und Wissen verfügt, aktuelle Desinformation auch tatsächlich zu identifizieren. Deren Gefahr liegt aber natürlich vor allem darin, dass MediennutzerInnen sie nicht als solche erkennen.

Das Besondere dieses Typs von Desinformation liegt in seinem Aktualitätsbezug. Damit ist gemeint, dass sowohl der Neuigkeitswert einer Information behauptet wird als auch deren gesellschaftliche Wichtigkeit. Aktuelle Desinformation tut also so, als sei sie ganz normaler Journalismus. Sie simuliert, dass sie dem Bürger genauso wie eine journalistische Nachricht dabei hilft, sich in einer komplexen Gesellschaft zu orientieren. Wie selbstverständlich erhebt sie daher auch einen Wahrheitsanspruch für ihre Faktendarstellung. Da aktuelle Desinformation zudem die Darstellungsweisen des Journalismus übernimmt, um ihre Glaubwürdigkeit zu steigern, kann man sie von einer journalistischen Nachricht im Prinzip nicht unterscheiden – denn dass sie falsch ist, kann man ihr ja nicht unmittelbar ansehen. Falsch ist die aktuelle Desinformation nämlich immer. Ihr irreführendes Potential kann sie nur entfalten, wenn sie tatsächlich unzutreffende Tatsachenbehauptungen aufstellt.

Die vier Gründe, warum Fake-News erstellt werden:

1. Grund: Falschmeldungen werden aus Spaß verbreitet, um einen Scherz zu machen.

2. Grund: Die Urheber wollen durch „Clickbaiting“ Geld verdienen. Mit entsprechenden Sätzen wie „Das glaubst du nicht“ oder „So was haben Sie noch nie gesehen“, werden User animiert, auf die Meldung zu klicken. Das bringt den Urhebern Einnahmen ein. Und auch Werbevideos können durch manipulierte Bilder oder falsche Experten falsche Meldungen beinhalten, die aufgrund der seriösen Aufmachung dann erst mal geglaubt werden.

3. Grund: Es handelt sich um Verschwörungstheorie, etwa wie die erste Mondlandung 1969, die in Wahrheit eine Fake News sei. Als „Beweis“ wird ein Video angeführt, das vor dem Mondflug in Studio entstanden sein soll.

4. Grund: Die politische Meinung soll beeinflusst und so in eine gewünschte Richtung gelenkt werden. Derart politisch motiviert werden gezielt Gerüchte gestreut und geschickt Stimmung gegen Politik und Medien gemacht. Im Beispiel von Umweltschutz sind seit Jahren Akteure im Spiel, die Falschmeldungen verbreiten, zum Beispiel Shell beim Klimawandel, Think Tanks gegen die Klimaforschung oder etwa die „107 Lungenärzte“, die sich in der Diesel-Feinstaub-Debatte hinter die falschen Berechnungen von Dieter Köhler stellten.
Bis jetzt trifft unsere Definition auch auf einen bloßen journalistischen Irrtum zu. Die aktuelle Desinformation ist zusätzlich aber auch unwahrhaftig, das heißt sie wird im Wissen um ihre Falschheit in Umlauf gebracht. Eine mögliche Irreführung der Nutzer erfolgt somit nicht unbewusst, sondern wissentlich. Dabei ist keineswegs vorausgesetzt, dass aktuelle Desinformation direkt von einem unwahrhaftigen Kommunikator bezogen wird. Wissentlich in die Welt gesetzte Falschmeldungen werden auch von Menschen weiterverbreitet, die irrtümlicherweise von deren Wahrheit ausgehen. Aufgrund ihres Ursprungs betrachten wir sie aber weiterhin als aktuelle Desinformation.
Damit können wir aktuelle Desinformation als Kommunikation wissentlich und empirisch falscher Informationen zu neuen und relevanten Sachverhalten mit dem Anspruch auf Wahrheit definieren. Was sicherlich überrascht: Aktuelle Desinformation ist nicht notwendigerweise mit der Absicht zu täuschen verknüpft. Dies trifft nur auf propagandistische Desinformation zu, deren Urheber die Vorstellungen der Menschen manipulieren wollen, um ihre politischen Ziele zu erreichen. Sogenannte Clickbait-Desinformationen bedienen sich dagegen falscher Informationen nur, um Werbeeinnahmen zu erzielen. Die reißerischen Überschriften solcher Angebote dienen als Köder (bait), um die Neugier der Mediennutzer zu wecken und für Aufmerksamkeit in Form von Clicks und damit klingende Münze zu sorgen. Ob diese Falschmeldungen geglaubt werden oder nicht, ist den Produzenten gleichgültig. Die Wirkung dagegen ist potenziell dieselbe wie die von propagandistischer Desinformation.

**Aktuelle Desinformation und die Desinformationsordnung.**

Die öffentliche Debatte um aktuelle Desinformation dreht sich zumeist um die problematische Verbreitung von offensichtlichen Unwahrheiten. Aus dieser Problemsicht geht es zuvorderst darum, der Wahrheit wieder zu ihrem Recht zu verhelfen. So überprüfen Faktenchecker dubiose Nachrichten im Netz, bewerten ihren Wahrheitsgehalt und korrigieren sie. Diese Maßnahme ist auf den ersten Blick plausibel, scheint aber kaum geeignet, das eigentliche Problem zu lösen, mit dem wir es zu tun haben.

Das Problem liegt dabei weniger in den Urhebern von politischer Desinformation, die es immer geben wird. Es besteht vielmehr darin, dass aktuelle Desinformationen von nicht wenigen Menschen geglaubt werden. Wie kann man sich das erklären? Eine Antwort lautet: Aktuelle Desinformationen werden vor allem deshalb für wahr gehalten, weil sie einer gänzlich anderen Sicht auf unsere Gesellschaft und deren Institutionen wie Politik, Wissenschaft und Medien entsprechen. Es geht hier mithin gar nicht um die übliche Unterscheidung von wahr und falsch – es geht vielmehr allein darum, ob eine Informationsquelle als vertrauenswürdig oder eben nicht vertrauenswürdig angesehen wird.

Um das nachzuvollziehen, lohnt ein Perspektivenwechsel. Aus der Sicht normaler MediennutzerInnen sehen aktuelle Desinformationen genauso aus wie journalistische Nachrichten: Keiner Information kann man ansehen, ob sie falsch oder richtig ist – auch nicht der Nachricht aus der Tageszeitung. Dass man diese dennoch glaubt, hat mit Vertrauen zu tun. Um unseren Alltag zu meistern, verlassen wir uns auf ExpertInnen und gehen davon aus, dass diese kompetent und aufrichtig genug sind, um unsere Erwartungen zu erfüllen. Ohne dieses Vertrauen wären wir schlicht aufgeschmissen, denn wirklich wissen können wir nicht, ob die Diagnose der Ärztin zutrifft, die Kinder in der Kita gut aufgehoben sind, die journalistische Information wirklich relevante Orientierung bietet. Der Trick beim Vertrauen ist: Wir kompensieren unser mangelndes Wissen in diesen Bereichen durch das Wissen über deren Vertreter: die ÄrztInnen, die ErzieherInnen, die Presse usw. Auf die Nachrichtenmedien bezogen heißt das also: Wir halten eine Nachricht vor allem deshalb für wahr, weil wir dem Urheber dieser Nachricht vertrauen – nicht, weil sie wahr ist.
Was aber passiert, wenn dieses Vertrauen schwindet, gar in Misstrauen umkippt? Dann wenden sich die Menschen von den vormaligen ExpertInnen ab und suchen nach Alternativen. Wenn sie den etablierten Nachrichtenmedien nicht mehr vertrauen, gehen sie ins Netz und suchen dort nach Informationen, die ihnen eine andere Art der Orientierung bieten. Schon allein der Umstand, dass diese der Sicht der traditionellen Medien widersprechen, erhöht dann die Wahrscheinlichkeit, dass sie geglaubt werden. Wenn die Bürger der Politik nicht mehr vertrauen, suchen sie auch im politischen Bereich nach Alternativen. Informationen, die ihre skeptische Sicht auf die etablierten Parteien bestätigen, werden dann schon allein deshalb eher geglaubt. In beiden Fällen profitieren die Urheber aktueller Desinformation also von schwindendem oder gar verlorenem Vertrauen in gesellschaftliche Institutionen wie Politik und Medien. Wäre das Vertrauen bei allen BürgerInnen weiterhin hoch, hätte aktuelle Desinformation deutlich weniger Chancen.

Die drei Arten von Desinformation:

1. Nachrichten, die einem bestimmten Thema übertriebene Aufmerksamkeit widmen

Der falsche Eindruck wird erweckt, dass ein Thema relevanter ist, als es sein sollte. Nicht das Thema an sich, sondern die vermeintliche Relevanz ist "fake". Eine unveröffentlichte Studie der Harvard Universität zeigt, wie die US-Medien überproportional häufig über Hillary Clintons vermeintlichen E-Mail-Skandal berichteten.

2. Propaganda

Ist klassischer Bestandteil von Politik und Wahlkämpfen und beschreibt das Vermischen von wahren und falschen Informationen, um die andere Seite zu schwächen und die eigene zu stärken.

3. Gezielte Desinformation

Dabei handelt es sich oft um absichtlich frei erfundene, als Nachrichten getarnte Geschichten. Die behaupten dann zum Beispiel, dass Angela Merkel bewusst ISIS in Europa operieren lässt. Dies geschieht nicht immer aus politischen, sondern zum Teil einfach aus finanziellen Gründen. Fake News sollen Menschen beeindrucken, damit die Leser diese anklicken, liken und größtmöglich weiterleiten. Durch Werbeschaltung auf diesen Seiten wird Geld verdient und betrügerische Interessen, wie durch das sogenannte "phishing" – also das Ausspähen persönlicher Daten und Passwörter – verwirklicht.

**Was tun gegen Desinformation?**

Was kann mir helfen, wenn ich durch den Social Media Feed scrolle und nicht bei jeder Nachricht eine lange Hintergrundrecherche durchführen möchte? In dieser Lerneinheit gehen wir von acht Merkmalen aus, anhand derer man Fake-News gut erkennen kann:

1. Merkmal: Der Schreibstil ist häufig emotional, sensationell, stellt viele Frage und verwendet häufig Ausrufezeichen.
2. Merkmal: Wer ist der Autor? Ein fehlender Urheber ist oft ein Hinweis auf Fake News. Auch ein Blick ins Impressum ist hilfreich. Laut Gesetz muss jeder Betreiber einer Webseite eine vollständige Adresse angeben. Wird das Thema auf anderen Seiten ähnlich aufgearbeitet, oder erscheint es sogar exakt im gleichen Wortlaut? Kopierte Inhalte deuten auf unseriöses Copy & Paste-Verhalten hin. Texte, die verkürzt nur auf Schlagzeilen beruhen, wenig Inhalt und viel Meinung präsentieren, sollten mit Vorsicht genossen werden.
3. Merkmal: Woher stammt die Meldung, wo hat sie ihren Ursprung? Gib dazu eine Passage der Nachricht in die Suchmaschine ein und schau mal, was dir angezeigt wird.
4. Merkmal: Die Quelle der Nachricht. Such diese selber raus und vergleiche die Angaben. Du kannst für deine Recherche auch die Webseiten andere Länder mit einbeziehen.
5. Merkmal: Gleiche Zahlen, Daten und Fakten und Studien miteinander ab. Stimmen die genannten Zahlen und Originalmeldungen überein spricht es für den Wahrheitsgehalt einer Meldung.
6. Merkmal: Aktualität, aus welcher Zeit stammt die Information? In Suchmaschinen hilft ein voreingestellter Filter, der den Zeitraum der Ergebnisse einschränkt, die neusten Informationen zu einem Thema zu finden.
7. Merkmal: Suche nach den im Artikel verwendeten Bildern. Mach einen Screenshot und gib ihn zur Bildersuche ein. Noch einfacher geht es mit Webseiten wie TinEye. Hier kannst du das Bild hochladen, die Seite untersucht dann den Ursprung der Bildquelle.
8. Merkmal: Schau bei Recherche-Webseiten vorbei, hier arbeiten Journalisten und Redakteure täglich daran, Fake News aufzudecken: Mimika.at unterstützt als z.B. als Verein zur Aufklärung über Internetmissbrauch den Kampf gegen falsche Behauptungen. Die Organisation newsquardtech.com arbeiten mit einem Team aus Journalisten daran, Nachrichten professionell zu untersuchen und zu bewerten, lesenswert etwa der Misinformation Monitor.

Sie haben nun viele Informationen über Fake News erhalten und gleichzeitig acht Merkmale mit an die Hand bekommen, um Fake News von ‚Real News‘ unterscheiden zu können. Doch wozu sollten Sie das alles lernen?

Das Wissen um Fake News ist wie ein Werkzeugkasten, mit dem wir Nachrichten und Überzeugungsversuche anderer Art evaluieren können. Auf der nächsten Seite finden Sie daher noch einmal eine vereinfachte Übersicht über die Merkmale von Fake News, die Sie in dieser Lerneinheit kennengelernt haben.

**S5 Fake News Texts (German)**

***Politically Neutral***

**GEZ-Urteil: Verfassungsrichter und Urheber des Rundfunkbeitrags sind Brüder.**

Am Freitag (18. März 2016) entschied das Bundesverwaltungsgericht in Leipzig, dass der Rundfunkbeitrag von der Gebühreneinzugszentrale GEZ ohne ein Empfangsgerät rechtens ist. Den abgeschmetterten Bürgern bleibt nur noch, Beschwerde beim Bundesverfassungsgericht in Karlsruhe einlegen.

Aber da stoßen sie auf einen Familienbetrieb. Schon im Mai 2013 hat der dortige Vizepräsident Ferdinand Kirchhof das GEZ-Gesetz über den Zwangs-Rundfunkbeitrag seines Bruders durchgewunken. Wie jedermann weiß, ist Blut dicker als Wasser. Paul Kirchhof (73) wurde übrigens 1981 als Professor und Inhaber eines Lehrstuhls für Staatsrecht an die Universität Heidelberg berufen. Die Uni Heidelberg gehört dem Land Baden-Württemberg, ist also öffentlich-rechtlich – genauso wie ARD, ZDF und Deutschlandradio. Die öffentlich-rechtlichen Sender bestellten zum Erstellen des GEZ-Gesetzes demnach ein Gutachten eines öffentlich-rechtlichen Professors. Kopp-Autor Wisnewski fragt die Leser: “Haben Sie sich nicht schon mal darüber gewundert, dass praktisch jede Klage gegen die neue Haushaltsabgabe vor dem Bundesverfassungsgericht scheitert?“

**Forscher publizieren Studie: Elektromagnetische Wellen erzeugen Krebs.**

Eine Gruppe von Forschern der Universität Peking haben angesichts der Corona-Krise eine Studie zur Wirkung elektromagnetischer Wellen auf menschliche Zellen veröffentlicht.

Um die Wirkung elektromagnetischer Hochfrequenz-Strahlung (EMR), wie sie bei Mobilfunkwellen und vor allem bei 5G-Wellen vorkommt, zu untersuchen, wurden Zellen mit einer Strahlung von 1800 MHz beschossen. Dabei wurde die Immunfluoreszenz der Zellen gemessen und Bilder der Struktur der Mitochondrien, die direkt den Zelltod (Apoptose) widerspiegelten, mit dem Elektronenmikroskop aufgenommen.

Die Ergebnisse zeigten, dass die Lebensfähigkeit der Zellen nach 48 Stunden stark vermindert war. Weitere epidemiologische Studien haben gezeigt, dass EMR Hirntumore, Brustkrebs, Nebennieren- und Herztumore verursachen kann.

***Politically Right***

**Gericht bestätigt: AfD-Mitglieder jetzt offiziell „alle“ Nazis!**

Das Amtsgericht hatte es abgelehnt, ein Strafverfahren wegen des Verdachts der Beleidigung gegen ein Mitglied der Partei Bündnis 90/Die Grünen durchzuführen. Der Beschwerdeführer warf dem beschuldigten Grünenpolitiker vor, dass dieser ihn anlässlich einer Wahlkampfveranstaltung für die Landtagswahl Baden-Württemberg wiederholt als „Nazi“ beschimpft habe. Die Begründung des Gerichts zur Abweisung der Klage wurde direkt aus der grünen Nazi-Tonne gezogen: Ein Fall der grundsätzlich strafbaren Schmähkritik könne nicht angenommen werden, denn der Grünenpolitiker habe sich von der Partei AfD wegen ihres Programmes, ihres Auftretens und des Duldens rechtsradikaler Parteimitglieder bei gleichzeitigem Beanspruchen der bürgerlichen Mitte abgrenzen wollen.

Der oder die Richter haben anscheinend keine Ahnung mehr, was „Nazi“ bedeutet.

Was für das eine AfD-Mitglied gilt, gilt, interpretiert man das Urteil so wie ich, für alle AfD-Mitglieder. AfD-Mitglieder sind nun also „alle“ Nazis.

**Schon gewußt? – dass Krebs in Israel eine Ausnahmeerscheinung ist? Warum das so ist.**

Die nachfolgende Meldung sollte man im Zusammenhang mit den Aussagen von Dr. Hamer sehen und mit der Annahme, dass die Krebserkrankungen von heute durch vielfältige Attacken entstehen. Krebserkrankungen und Tode kommen in Israel außergewöhnlich selten vor, und der Trend der wenigen Erkrankungen ist weiterhin rückläufig. So starben in Israel im Jahr 2003 160 Menschen und im Jahr 2004 152 Menschen an Krebs. Was sind die wahren Gründe dafür, dass der Bund aus Schulmedizin, Pharmaindustrie und Politik bei der nicht-jüdischen Bevölkerung der Welt stur wie ein Panzer an ihrem System Chemotherapie + Bestrahlung + Chirurgie + Morphiumgabe festhält?

Die Antwort scheint klar zu sein: Bei dieser Form der Behandlung von Krebserkrankungen handelt sich um eines der gigantischsten Geschäfte aller Zeiten. Das könnte der Hauptgrund für das ansonsten nicht nachvollziehbare Desinteresse an wirkungsvollen, ganzheitlichen Therapieformen sein.

***Politically Left***

**Tönnies versucht durch 15 neue GmbH-Gründungen das Werkverträgeverbot auszuhebeln.**

Nach dem Coronaskandal in der Fleischfabrik von Großunternehmer Clemens Tönnies wurden scharfe Auflagen für sein und ähnliche Unternehmen diskutiert. Ab dem 1. Januar 2021 wird es ein Verbot von Werkverträgen für Großunternehmen geben. Ausgenommen hiervon sind nur Unternehmen des Fleischerhandwerks mit bis zu 49 tätigen Personen. Von Tönnies 16.500 Mitarbeitern sind bisher die Hälfte über Werkverträge mit Subunternehmern angestellt, die für die Arbeitenden in schlechten Arbeits- und Lohnbedingungen resultieren. Das Werkverträgeverbot soll das ändern, doch Tönnies scheint etwas dagegen zu haben. Kürzlich hat er 15 neue GmbHs gegründet, um das Werkverträgeverbot zu umgehen. Wenn die Mitarbeiter in kleineren Unternehmen angestellt sind, kann er sie auf mehrere GmbHs aufteilen und so die Verträge mit den Subunternehmern behalten.

**Die weltweite Benachteiligung von Frauen nimmt ungeheure Ausmaße an.**

Dass Frauen in der Gesellschaft benachteiligt werden, ist nichts neues. Neue Zahlen zeigen aber, wie weitreichend diese Benachteiligung auf der gesamten Welt verteilt ist. Frauen werden unterdrückt, als weniger werthaft als Männer angesehen und machen doch mehr Arbeit als alle Männer zusammen. Die neuesten Zahlen bestätigen das: Frauen sind 51 Prozent der Population, aber 70 Prozent der Armen und 83 Prozent aller Alleinerziehenden, machen 66 Prozent der Arbeit, produzieren 50 Prozent des Essens, aber verdienen nur 11 Prozent des Lohns und nur 1 Prozent des Landes.

Wer immer noch nicht glaubt, dass Frauen benachteiligt werden, braucht nur auf diese Zahlen zu schauen. Im Kampf gegen die globale Ungerechtigkeit ist es wichtig, die Geschlechter endlich gleichzustellen. Wer diesen Zahlen nicht glaubt, der hat immer noch nicht erkannt, dass Frauen in der Gesellschaft benachteiligt werden.

**S6 Real News Texts (German)**

***Politically Neutral***

**Vorbehalte gegen Peter Müller.**

Der beabsichtige Wechsel von Peter Müller ans Bundesverfassungsgericht bringt die Frage auf: Ist es gut für die Demokratie, wenn ein amtierender Ministerpräsident an das höchste deutsche Gericht wechselt. Die FDP findet: Nein.

Der Vorsitzende der FDP-Fraktion im Hessischen Landtag, Florian Rentsch, schrieb Müller einen Brief, der der Süddeutschen Zeitung vorliegt. Darin heißt es, die "große Akzeptanz" des Bundesverfassungsgerichts entspringe auch der Tatsache, dass die Richter dort "grundsätzlich nicht aus der ersten Reihe der aktiven Politik stammen". Werde diese Praxis nun geändert, könne dies dazu führen, das öffentliche Vertrauen in das Karlsruher Gericht "als selbstständigem Verfassungsgericht erheblich zu beschädigen". In Karlsruhe ist zwar nicht nur rechtliche Theorie, sondern auch politische Praxis gefragt, nicht nur rechtliche Akribie, sondern auch diplomatisches Geschick. Das kann Peter Müller helfen. Trotzdem wird er lange souverän und unabhängig urteilen müssen, bis die Bürger ihr naheliegendes Vorurteil gegen ihn ablegen.

**Pharmaindustrie hat weiter freie Hand.** Die Enthüllungen von SZ/NDR/WDR über Anwendungsbeobachtungen (AWB) für Medikamente beschäftigen auch die Politik. Die Bundesregierung hat jetzt auf eine parlamentarische Anfrage der Linken-Fraktion geantwortet. Darin verteidigt sie die bisherige Praxis. Die Grünen wollen dagegen eine Änderung des Arzneimittelgesetzes. Experten fordern, dass Patienten künftig zumindest informiert werden, ob sie in einer AWB beobachtet werden. Im März hatte ein großer Rechercheverbund aufgedeckt, welches Ausmaß die Ärzte-Korruption bei sogenannten „Anwendungsbeobachtungen“ erreicht hat. Demnach beteiligt sich jeder zehnte Arzt an Scheinstudien. Offiziell dienen diese AWBs dazu, wissenschaftliche Erkenntnisse darüber zu sammeln, wie gut Patienten bestimmte Medikamente vertragen. Tatsächlich ermöglicht diese Praxis der Pharmaindustrie aber, teure und nicht selten überflüssige Medikamente in den Markt zu drücken, weil die Ärzte für die „Beobachtung“ ihrer Patienten ein oft hohes Honorar bekommen.

***Politically Right***

**Seehofer sieht „jederzeit Gefahr“ von Anschlägen in Deutschland.**

Angesichts der Terroranschläge in Dresden, Paris, Nizza und Wien, bei denen in den vergangenen Wochen neun Menschen getötet worden sind, hat sich der Bundestag am Donnerstag in einer aktuellen Stunde mit der Bekämpfung des islamistischen Terrors in Europa befasst. Bundesinnenminister Horst Seehofer warnte gleich zum Auftakt der Debatte, mit Anschlägen müsse jederzeit gerechnet werden. Er schloss sich damit Verfassungsschutzpräsident Thomas Haldenwang an, der am Mittwochabend gewarnt hatte, die Sicherheitsbehörden in Deutschland müssten derzeit „sehr wachsam“ sein. Jetzt gelte es, „einen sehr scharfen Blick auf die uns bekannten Gefährder“ zu werfen. Es gebe sicherlich den oder die ein oder andere, die über Nachahmungstaten nachdächten. Laut Seehofer sind den Sicherheitsbehörden aktuell 615 islamistische Gefährder in Deutschland bekannt. 217 von ihnen seien Deutsche, 119 hätten eine deutsche und eine weitere Staatsbürgerschaft, 273 seien Ausländer, so Seehofer.

**Bauen, bis Biden kommt.**

Israel schafft Fakten, solange Donald Trump noch im Weißen Haus ist. Jerusalem wird vom palästinensischen Westjordanland abgeschnitten und faktisch Israel einverleibt. Der Bau der seit Jahren immer wieder von Israel ins Spiel gebrachten Siedlung Givat Hamatos galt bis dahin als eine der roten Linien der internationalen Gemeinschaft. Jetzt haben die israelischen Behörden das Ende der Ausschreibung auf den 18. Januar 2021 festgelegt: also auf zwei Tage vor Amtsantritt des neuen Präsidenten Joe Biden. In diesem Zusammenhang glaubte kaum jemand an einen Zufall, als Israel vor einer Woche den Ausbau einer weiteren Siedlung in Ostjerusalem genehmigte – und zwar in Ramat Shlomo, just jener Siedlung, deren Ausbau Ministerpräsident Benjamin Netanjahu während des Besuchs von Biden 2010 in Jerusalem genehmigte. Dies hatte damals zu der ersten tiefen Verstimmung zwischen Netanjahu und Washington geführt, das daraufhin einen vorübergehenden Stopp des weiteren Siedlungsausbaus erzwang.

***Politically Left***

**Polizei durchsucht Zeitarbeitsfirmen.**

Die Bundesregierung hat versprochen, entschieden gegen die Missstände in Schlachtbetrieben vorzugehen. Nun haben 800 Beamte die Geschäftsräume von Subunternehmen und Wohnunterkünfte durchsucht. Die ohnehin unter besonderer Beobachtung stehende Fleischindustrie hat ihren nächsten Skandal. Laut Bundespolizei steht ein "Konstrukt aus verschiedenen Zeitarbeitsfirmen" im Fokus, über das die zehn Hauptbeschuldigten im vergangenen halben Jahr mindestens 82 Menschen mit falschen Dokumenten oder als "Scheinstudenten" nach Deutschland geschleust haben sollen.

Die Ermittler gehen davon aus, dass mit dem "strafbaren Geschäftsmodell" rund 1,5 Millionen Euro erwirtschaftet wurden.

**Die Geschichte der ungerechten Bezahlung.**

Dass Frauen weniger verdienen als Männer, ist eine wirtschaftliche Tatsache. 21 Prozent beträgt die geschlechtsspezifische Lohnlücke in Deutschland.

Bis heute ist der Gender Pay Gap in allen Ländern Wirklichkeit: Besonders weit klaffen die Gehälter im weltweiten Vergleich in Südafrika, Südkorea oder Pakistan auseinander, mit jeweils deutlich mehr als 30 Prozent. Weniger groß ist der Abstand in Belgien, Griechenland oder Costa Rica mit weniger als zehn Prozent. In Deutschland ist der Pay Gap zwischen den Geschlechtern im europäischen Vergleich mit 21 Prozent besonders hoch. Bei Frauen und Männern mit vergleichbaren Qualifikationen in einer ähnlichen Tätigkeit seien es zwar nur sechs Prozent Lohndifferenz, sagt Katharina Wrohlich vom Deutschen Institut für Wirtschaftsforschung (DIW). Trotzdem findet sie, dass diese 21 Prozent – der sogenannte unbereinigte Gender Pay Gap – aussagekräftig seien.

**S7 Fake News Texts (English translation)**

***Politically Neutral***

**GEZ ruling: Constitutional judge and originator of the broadcasting fee are brothers.** On Friday (March 18, 2016), the Federal Administrative Court in Leipzig ruled that the broadcasting fee from the GEZ fee collection agency is legal without a broadcasting device. As a last resort, the shot down citizens can only appeal to the Federal Constitutional Court in Karlsruhe.

But there they run into a family business. Back in May 2013, the vice president there, Ferdinand Kirchhof, waved through his brother's GEZ law on compulsory broadcasting fees. As everyone knows, blood is thicker than water. Incidentally, Paul Kirchhof (73) was appointed professor and chair of constitutional law at the University of Heidelberg in 1981. The University of Heidelberg belongs to the state of Baden-Württemberg, so it is a public institution – just like ARD, ZDF and Deutschlandradio. The public broadcasters ordered an expert opinion of a public law professor for the creation of the GEZ law. Kopp author Wisnewski asks Kopp-readers: “Haven't you ever wondered why practically every complaint against the new household tax fails before the Federal Constitutional Court?”

**Researchers publish study: electromagnetic waves cause cancer.** A group of researchers from Peking University have published a study on the effect of electromagnetic waves on human cells in light of the Corona Crisis.

To study the effect of electromagnetic radiofrequency radiation (EMR), as found in mobile radiation and especially 5G radiation, cells were bombarded with 1800 MHz radiation. The immunofluorescence of the cells was measured and images of the structure of mitochondria, which directly reflected cell death (apoptosis), were taken with an electron microscope.

The results showed that cell viability was greatly reduced after 48 hours. Further epidemiological studies have shown that EMR can cause brain tumors, breast cancer, adrenal tumors and heart tumors.

***Politically Right***

**Court confirms: AfD members now officially “all” Nazis!** The district court had refused to conduct criminal proceedings on suspicion of insulting a member of the Alternative of Germany (AfD) against a member of the Alliance 90/The Greens party. The complainant accused the accused Green politician of repeatedly insulting him as a “Nazi” on the occasion of an election campaign event for the Baden-Württemberg state elections. The court's reasoning for dismissing the complaint was drawn directly from the Green Nazi garbage can: a case of defamatory criticism, which is in principle punishable by law, could not be assumed, because the Green politician had wanted to distinguish himself from the AfD party because of its program, its appearance and the toleration of radical right-wing party members while at the same time claiming the middle-class center.

The judge or judges apparently no longer have any idea what “Nazi” means.

What applies to the one AfD member applies, if one interprets the ruling as I do, to all AfD members. AfD members are now “all” Nazis.

**Did you know? - that cancer is an exceptionally rare phenomenon in Israel? Why this is so.** The following message should be seen in connection with the statements of Dr. Hamer and with the assumption that the cancers of today are caused by multiple attacks. Cancer incidence and deaths are exceptionally rare in Israel, and the number of cancer cases continues to decline. For example, 160 people died of cancer in Israel in 2003 and 152 in 2004. What are the real reasons that the confederation of conventional medicine, pharmaceutical industry and politics stubbornly clings like a tank to their system of chemotherapy + radiation + surgery + morphine administration among the non-Jewish population of the world?

The answer seems clear: This form of cancer treatment is one of the most gigantic deals of all time. This could be the main reason for the otherwise incomprehensible lack of interest in effective, holistic forms of therapy.

***Politically Left***

**Tönnies tries to circumvent the ban on work contracts by founding 15 new limited liability companies.** After the Coronas scandal at the meat factory of big businessman Clemens Tönnies, tough requirements for his and similar companies were discussed. From January 1, 2021, there will be a ban on work contracts for large companies. Only companies in the butcher's trade with up to 49 employees will be exempt. Of Tönnies' 16,500 employees, half have so far been employed via contracts for work and services with subcontractors, resulting in poor working and wage conditions for the workers. The ban on work contracts is supposed to change that, but Tönnies seems to have something against it. He recently set up 15 new GmbHs to get around the ban on work contracts. If the employees are employed by smaller companies, he can split them up among several GmbHs and thus keep the contracts with the subcontractors.

**The worldwide discrimination against women is assuming immense proportions.** That women are disadvantaged in society is nothing new. But new figures show how widespread this discrimination is throughout the world. Women are oppressed, seen as less valuable than men, and yet they do more work than all the men put together. The latest figures confirm this: women are 51 percent of the population but 70 percent of the poor and 83 percent of all single parents, do 66 percent of the work, produce 50 percent of the food, but earn only 11 percent of the wages and only 1 percent of the land.

Anyone who still doesn't believe that women are disadvantaged need only look at these numbers. In the fight against global injustice, it is important to finally put the sexes on an equal footing. Anyone who does not believe these figures has still not recognized that women are disadvantaged in society.

**S8 Real News Texts (English translation)**

***Politically Neutral***

**Objections to Peter Müller.** Peter Müller's intended move to the Federal Constitutional Court raises the question: Is it good for democracy if a sitting prime minister moves to Germany's highest court. The FDP thinks: No.

The chairman of the FDP parliamentary group in the Hessian state parliament, Florian Rentsch, wrote Müller a letter that was obtained by the Süddeutsche Zeitung. It says that the “great acceptance” of the Federal Constitutional Court also stems from the fact that the judges there “are basically not from the first row of active politics”. If this practice were now changed, it could lead to considerable damage to public confidence in the Karlsruhe court “as an independent constitutional court”. In Karlsruhe, not only legal theory but also political practice is required, not only legal meticulousness but also diplomatic skill. That can help Peter Müller. Nevertheless, he will have to judge sovereignly and independently for a long time until the citizens discard their natural prejudice against him.

**Pharmaceutical industry continues to have a free hand.** The revelations by SZ/NDR/WDR about observational studies (AWB) for medications are also occupying politicians. The German government has now responded to a parliamentary question from the Left Party. In it, it defends the current practice. The Greens, on the other hand, want an amendment to the German Medicines Act. Experts are calling for patients to at least be informed in the future whether they are being monitored in an AWB. In March a large research group had uncovered which extent the physician corruption reached with so-called “application observations”. According to this, every tenth physician participates in sham studies. Officially, these AWBs serve to gather scientific knowledge about how well patients tolerate certain drugs. In fact, however, this practice enables the pharmaceutical industry to push expensive and often superfluous drugs onto the market, because doctors often receive a high fee for “observing” their patients.

***Politically Right***

**Seehofer sees "danger at any time" of attacks in Germany.** In view of the terrorist attacks in Dresden, Paris, Nice and Vienna, in which nine people have been killed in recent weeks, the Bundestag held a topical debate on Thursday on how to combat Islamist terror in Europe. Right at the start of the debate, German Interior Minister Horst Seehofer warned that attacks must be expected at any time. In doing so, he echoed the words of Thomas Haldenwang, President of the Office for the Protection of the Constitution, who had warned on Wednesday evening that the security authorities in Germany must be “very vigilant” at present. Now it is necessary to take a “very sharp look at the dangerous persons known to us”. There are certainly one or two who are thinking about committing copycat acts. According to Seehofer, the security authorities are currently aware of 615 Islamist threats in Germany. 217 of them are Germans, 119 have German and another citizenship, and 273 are foreigners, Seehofer said.

**Building until Biden arrives.** Israel is creating facts while Donald Trump is still in the White House. Jerusalem is cut off from the Palestinian West Bank and de facto annexed to Israel. The construction of the Givat Hamatos settlement, which Israel has repeatedly brought into play for years, was until then considered one of the red lines of the international community. Now the Israeli authorities have set the end of the tender for January 18, 2021: that is, two days before the new President Joe Biden takes office. In this context, hardly anyone believed it was a coincidence when a week ago Israel approved the expansion of another settlement in East Jerusalem – in Ramat Shlomo, the very settlement whose expansion Prime Minister Benjamin Netanyahu approved during Biden's 2010 visit to Jerusalem. This led to the first deep disagreement between Netanyahu and Washington, which then forced a temporary halt to further settlement expansion.

***Politically Left***

**Police search temporary employment agencies.** The federal government has promised to take decisive action against abuses in slaughterhouses. Now 800 officers have searched the business premises of subcontractors and residential accommodation. The meat industry, already under special scrutiny, has its next scandal. According to the Federal Police, the focus is on a “construct of various temporary employment agencies” through which the ten main suspects are alleged to have smuggled at least 82 people into Germany with false documents or as “bogus students” in the past six months.

Investigators assume that the “criminal business model” generated around 1.5 million euros.

**The story of unfair pay.** That women earn less than men is an economic fact. 21 percent is the gender pay gap in Germany.

To this day, the gender pay gap is a reality in all countries: In a global comparison, salaries differ particularly widely in South Africa, South Korea or Pakistan, with well over 30 percent in each case. The gap is less wide in Belgium, Greece or Costa Rica, at less than ten percent. In Germany, the pay gap between the sexes is particularly high in a European comparison at 21 percent. For women and men with comparable qualifications in a similar job, the pay gap is only six percent, says Katharina Wrohlich of the German Institute for Economic Research (DIW). Nevertheless, she believes that this 21 percent – the so-called unadjusted gender pay gap – is meaningful.

**S9 Results for IFT Identification and IFT Explanation Tasks**

In calculating a general IFT score, we deviated from Ricco (2007) and our preregistration. Due to the low reliability the two tasks had on their own (ω = .51 for the identification task, and ω = .70 for the explanation task), we calculated the general score which yielded a sufficient reliability (ω = .76). We furthermore argue that the general IFT score is a theoretically sounder measure because it merges two important components of fallacy identification: *seeing* that and *understanding* why an argument is fallacious. The results for IFT identification and IFT explanation scores analyzed separately yielded the same outcomes concerning the hypotheses, both in significance testing and in direction.

As expected in Hypothesis 1, participants in the informal fallacy group performed significantly better in the IFT identification task (*M* = 0.87, *SD* = 0.11) than participants in the fake news group (*M* = 0.78, *SD* = 0.15), *t*(114) = 4.02, *p* < .001, *d* = 0.75. Concerning the learning intervention’s impact on the IFT identification task, group condition explained a significant portion of variance in informal reasoning, *R*^2^ = .12, *F*(1, 114) = 16.15, *p* < .001.

Hypothesis 2 expected the effect of group condition on discernment between real and fake news to be mediated by informal reasoning. This seemed to be the case for the IFT identification task as the indirect effect differed significantly from zero i.e., 0.03 (*SE* = .01), 95% CI [0.01, 0.06]. The partially standardized effect size was 0.24 (*SE* = 0.09), 95% CI [0.09, 0.44].

Note that, after informal reasoning is partialized out, the direct effect of group condition on discernment between real and fake news is non-significant for the IFT identification task; estimate = -0.05 (*SE* = 0.03), 95% CI [-0.10, 0.001]. The partially standardized effect size was -0.37.

Concerning the IFT explanation task, results were similar and differed only in effect size. Participants in the informal fallacy group performed significantly better in the IFT explanation task (*M* = 0.48, *SD* = 0.22) than participants in the fake news group (*M* = 0.27, *SD* = 0.14), Welch *t*(97.62) = 6.12, *p* < .001, *d* = 1.14. Group condition explained a significant portion of variance in informal reasoning, *R*^2^ = .25, *F*(1, 114) = 37.46, *p* < .001.

Hypothesis 2 expected the effect of group condition on discernment between real and fake news to be mediated by informal reasoning. This seemed to be the case for the IFT identification task as the indirect effect differed significantly from zero i.e., 0.07 (*SE* = .02), 95% CI [0.04, 0.12]. The partially standardized effect size was 0.55 (*SE* = 0.14), 95% CI [0.28, 0.90].

Note that, after informal reasoning is partialized out, the direct effect of group condition on discernment between real and fake news is significant for the IFT identification task; estimate = -0.09 (*SE* = 0.03), 95% CI [-0.15, -0.03], *p* < .001. The partially standardized effect size was -0.68. This result is another indicator that the fake news group also enhanced real- and fake news discernment through a mechanism we did not measure.

**S10 Detailed Account of Procedure**

As noted in the manuscript, the study took part in two online sessions.

Session 1: In the first session, participants joined a *Zoom* meeting with their cameras activated. As *Zoom* was used in almost all courses at that time, all students were familiar with using it. The Zoom meeting involved 3 to 10 participants and an instructor (the first author of the present manuscript). During the learning intervention the instructor could check that participants were not in fact occupied with other activities besides the learning intervention. Participants were also allowed to ask questions in a breakout-room so that the other participants would not get distracted. In this first session, the participants worked through the learning materials and answered 5 of the learning intervention’s feedback questions (see Materials). When a participant was done with the learning intervention, they could leave the meeting. Participants were made aware of the fact that there were multiple groups – but not what differentiated these groups – and that they should not talk to their peers about the study.

Session 2: One week after they had completed the first session, they received a personalized link to participate in the second session. They had 3 days to work through that second session, otherwise the link would have been expired and they only would have received less course credit. Only one participant did not complete the second questionnaire in time. Participants worked on the materials in Session 2 unsupervised, as they would when reading news in real life. The second session consisted of a short revision (*M* = 78.30 seconds, *SD* = 41.88 seconds) of the questions testing informal fallacies or the characteristics of fake news. Afterwards two of Session 1’s feedback questions were shown. These were followed by four new feedback questions to further reactivate the learning intervention’s contents.

Participants worked through the IFT afterwards, following an instruction to answer spontaneously, with short texts only, and honestly. If they had no clue how to answer a question they were asked to answer intuitively. This instruction was given so that participants of the fake news group would not get frustrated if they did not know how to exactly name a certain fallacy.

After finishing the IFT, participants worked on the fake news discernment task. The order of the news articles was randomized for every participant. Finally, participants were asked to provide demographic information concerning age, gender, education level, native language. They were also asked to rate their political orientation. Another question asked them if they already saw one or more of the presented news before and if in fact they did, they had to indicate how many on a slider ranging from 1 to 12. This variable was unrelated to all other measures (*r*s ranging from -0.07 to .10).

For all tasks we asked the participants not to use any external information sources (e.g., google the news presented). The participants needed on average 66.26 minutes to finish both parts of the study (*SD* = 15.13, median = 62.66 minutes).

**S11 Additional Information on Data Analyses and Data Quality Checks**

There were two missing data points for two items in the fake news questionnaire (one for each item). Because the rest of the data from the two participants was correctly entered, we calculated the mean values for fake and real news accuracy without these scores.

We found one outlier in real and fake news discernment accuracy in the informal fallacy group and four outliers for the IFT identification scores (3 for the informal fallacy group, 1 for the fake news group). Outliers were winsorized according to the procedure reported in the Data Analysis section of the main manuscript.

For regression analyses, we checked several parameters: Cook’s distance was < 1 for all cases – including the multivariate outliers (Mahalanobis Distance > 10) – so we still included the multivariate outliers (Field, 2018). We also tested if deleting them would have had substantial effects on other regression coefficients or *R*^2^, which it did not. We did not find an indication for strong multicollinearity (*VIF*s = 1.000) and Durbinson-Watson test values ranged between 1.896-2.378 for all regressions. The randomly generated seed for *PROCESS*-bootstrapping was 295786.**S12 Deviations from Preregistration**

We deviated from our preregistration in a few ways: First, we changed the order of hypotheses as it seemed to make more sense to investigate the effect of the learning intervention on the mediator and the mediating effect before investigating the total effect. We preregistered the total effect as our first hypothesis, the effect of the learning intervention on informal reasoning as the second hypothesis, and the mediation effect as the third hypothesis.

Second, we did not conduct some preregistered exploratory analyses for the following reasons: for demographic variables and political ideology we did not conduct our preregistered exploratory analyses because potential findings would not be generalizable due to constraints in our sample. Concerning demographic variables, only 8 (of 58) participants in the fake news group – and 16 in the informal fallacy group – identified themselves as male and none as non-binary, excluding the possibility of any meaningful results concerning gender effects. Moreover, all participants indicated that their highest degree was a high-school degree, and only three people indicated that German was not their native language. Therefore, no exploratory analyses concerning demographic variables were conducted: results would very likely not be generalizable.

Concerning political ideology, only two participants indicated to be more right-leaning than left-leaning – ticking a value of 7 which is the first value after the mid of the 11-point scale we employed for measuring political ideology. Therefore, we also did not conduct any analyses on the influence of political ideology on news discernment: if our sample primarily consists of left-leaning people, variance in news accuracy ratings would very likely not be a result of variance in participant’s political ideology. Results for the other preregistered exploratory analyses can be found in S12.

Third, we employed more than the preregistered ways of excluding careless responders. We did so because answers in the open answer field of the IFT explanation task indicated that more people engaged in careless responding than we initially thought. The results did not change in significance due to these measures. There was, however, a slight increase in effect sizes.

Fourth, we deviated from our preregistered sample size. In our *aspredicted*-document we had indicated that we wanted to test 120 participants. After scanning the data for eligibility when we had collected data of 120 participants, we saw that data of less than 110 participants was eligible. Hence, we examined 11 participants more to make sure we had enough eligible data. Thus, our *N* amounted to 131 participants. **S 13 Exploratory Analysis**

***Learning Transfer***

This exploratory analysis was not preregistered and is therefore reported in the supplemental material. To investigate if teaching about certain types of informal fallacies only enhances IFT scores for those types of fallacies, we computed general IFT scores for IFT tasks whose fallacies were introduced in the informal fallacy intervention and those IFT tasks whose fallacies were not introduced, respectively. A mixed ANOVA (between subjects factor: experimental treatment; within-subjects factor: fallacy introduction) revealed a significant main effect of the experimental treatment, participants who were in the informal fallacy group scored better in the IFT tasks (*M* = 0.34, *SD* = 0.08) than participants in the fake news group (*M* = -0.34, *SD* = 0.08), F(1,114) = 32.44, *p* < .001, η^2^ = .22. Additionally, there was a significant interaction between experimental treatment and fallacy introduction, indicating that the treatment had a stronger effect on identifying and analyzing fallacies which were introduced in the learning intervention, *F*(1,114) = 9.19, *p* = .003, η^2^ = .08. A post hoc test showed that the informal fallacy group (*M* = 0.21, *SD* = 0.82) still performed better than the fake news group (*M* = -0.21, *SD* = 0.88) for IFT tasks which consisted in assessing informal fallacies which had not been introduced in the informal fallacy intervention, *t*(114) = 2.66, *p* = .009, *d* = 0.49. This effect was more pronounced, however, for fallacies which had been introduced in the learning intervention, Welch *t*(110.49) = 6.91, *p* < .001, *d* = 1.28. A graphical representation can be found in Figure S1.

**Figure S1.**

*Descriptive statistics with experimental treatment as between subject and fallacy introduction as within-subject variable.*

*
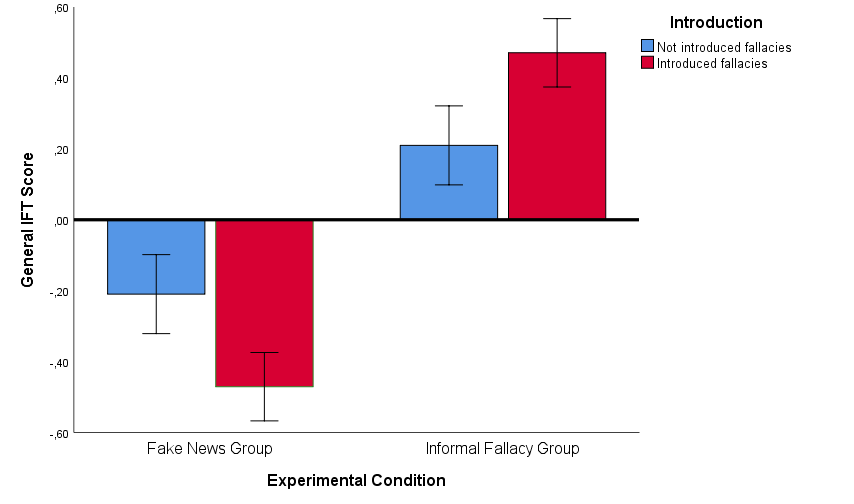
*

*Note.* Error bars show +/-1 *SE*.

**Supplement References**

Field, A. (2018). *Discovering statistics using IBM SPSS Statistics.* Sage.

Leiner, D. J. (2019). Too Fast, too straight, too weird: Non-reactive indicators for meaningless data in internet surveys. *Survey Research Methods, 13*(3), 229-248. https://doi.org/10.18148/SRM/2019.V13I3.7403

Maniaci, M. R. & Rogge, R. D. (2014). Caring about carelessness: Participant inattention and its effects on research. *Journal of Research in Personality, 48*, 61–83. https://doi.org/10.1016/j.jrp.2013.09.008

Meade, A. W. & Craig, S. B. (2012). Identifying careless responses in survey data. *Psychological Methods, 17*(3), 437–455. https://doi.org/10.1037/a0028085

Patton, J. M., Cheng, Y., Hong, M. & Diao, Q. (2019). Detection and treatment of careless responses to improve item parameter estimation. *Journal of Educational and Behavioral Statistics, 44*(3), 309–341. https://doi.org/10.3102/1076998618825116
